# Machine learning-identified stemness features and constructed stemness-related subtype with prognosis, chemotherapy, and immunotherapy responses for non-small cell lung cancer patients

**DOI:** 10.1186/s13287-023-03406-4

**Published:** 2023-09-07

**Authors:** Mingshan Liu, Ruihao Zhou, Wei Zou, Zhuofan Yang, Quanjin Li, Zhiguo Chen, Lei jiang, Jingtao Zhang

**Affiliations:** 1 Department of Thoracic Surgery, The First Affiliated Hospital of Nanchang University, Nanchang, 330006 Jiangxi China; 2 Jiangxi Hospital of China-Japan Friendship Hospital, National Regional Center for Respiratory Medicine Nanchang, Jiangxi, 330000 People’s Republic of China; 3 Department of Anesthesiology, West China Hospital, Sichuan University, Chengdu, 610041 Sichuan Province People’s Republic of China

**Keywords:** Non-small cell lung cancer, Cancer stem cell, Stemness index, Consensus clustering, Immunotherapy, Machine learning

## Abstract

**Aim:**

This study aimed to explore a novel subtype classification method based on the stemness characteristics of patients with non-small cell lung cancer (NSCLC).

**Methods:**

Based on the Cancer Genome Atlas database to calculate the stemness index (mRNAsi) of NSCLC patients, an unsupervised consensus clustering method was used to classify patients into two subtypes and analyze the survival differences, somatic mutational load, copy number variation, and immune characteristics differences between them. Subsequently, four machine learning methods were used to construct and validate a stemness subtype classification model, and cell function experiments were performed to verify the effect of the signature gene *ARTN* on NSCLC.

**Results:**

Patients with Stemness Subtype I had better PFS and a higher somatic mutational burden and copy number alteration than patients with Stemness Subtype II. In addition, the two stemness subtypes have different patterns of tumor immune microenvironment. The immune score and stromal score and overall score of Stemness Subtype II were higher than those of Stemness Subtype I, suggesting a relatively small benefit to immune checkpoints. Four machine learning methods constructed and validated classification model for stemness subtypes and obtained multiple logistic regression equations for 22 characteristic genes. The results of cell function experiments showed that *ARTN* can promote the proliferation, invasion, and migration of NSCLC and is closely related to cancer stem cell properties.

**Conclusion:**

This new classification method based on stemness characteristics can effectively distinguish patients' characteristics and thus provide possible directions for the selection and optimization of clinical treatment plans.

**Supplementary Information:**

The online version contains supplementary material available at 10.1186/s13287-023-03406-4.

## Introduction

Lung cancer is a malignant tumor originating from the bronchial mucosa or glands of the lung, with the fastest growing morbidity and mortality rate [1], and has become the leading cause of death among oncology patients [2]. Currently, non-small cell lung cancer (NSCLC) is mainly treated by a combination of surgery, supplemented by chemotherapy, radiotherapy, molecular targeted therapy, and immunotherapy according to tumor stage, pathological type, and the presence or absence of mutations in the driver genes [3]. Despite the efficacy of targeted therapy and immunotherapy for patients with advanced NSCLC in recent years, the 5-year survival rate for patients without susceptibility mutations has yet to be improved [4, 5]. Therefore, new diagnostic and therapeutic modalities for NSCLC are urgently needed to be explored.

Cancer stem cells are a very small number of tumor cells in tumor tissue with biological properties such as unlimited proliferation, self-renewal and multidirectional differentiation, which can form a heterogeneous series of cell populations in that tumor [6, 7]. It was found that cancer stem cells express a variety of ABC transporter proteins on their surface [8], which can effectively transport chemotherapeutic drugs to the extracellular thereby reducing the damage of chemotherapeutic drugs to cells; in addition, the strong repair ability of cancer stem cells can repair the damage to the maximum extent [9]. Therefore, the presence of lung cancer stem cells is one of the main reasons for the low survival rate of lung cancer patients, their strong resistance to conventional radiotherapy and chemotherapy, and their easy recurrence after surgery or radiotherapy treatment [10]. An in-depth study of NSCLC cell stemness could provide new ideas for the study of lung cancer occurrence, drug resistance, and metastasis and also bring new hope for the clinical treatment of lung cancer.

The immune microenvironment is a specific microenvironment on which cancer stem cells depend, and the immune infiltrating cells in it play an important role in the development and progression of NSCLC [11, 12]. It has been reported that in the immune microenvironment of lung cancer, the interaction of co-stimulatory molecules *CD40* and *CD154 *(*CD40L*) molecules on the surface of B cells resulted in an enhanced ability of activated B lymphocytes to present antigens to CTL cells, leading to a significant increase in the levels of *IFN-γ* and *IL-2* secreted by CTL cells and enhanced the killing effect of CTL on lung cancer stem cells [13]. As the regulatory relationship between immune infiltrating cells and cancer stem cells is gradually elucidated, improving the tumor immune microenvironment and disrupting the "protective" effect of the tumor microenvironment on cancer stem cells has become a new strategy for lung cancer treatment [14–16]. In addition, lung cancer immunotherapy targeting immunosuppressive signals, and immune checkpoints, is also being studied, and a large number of studies have shown that immune checkpoint inhibitors, represented by *PD-1/L1* and *CTLA-4*, can restore the antitumor immune response and play a role in killing tumors [17].

**Fig. 1:**
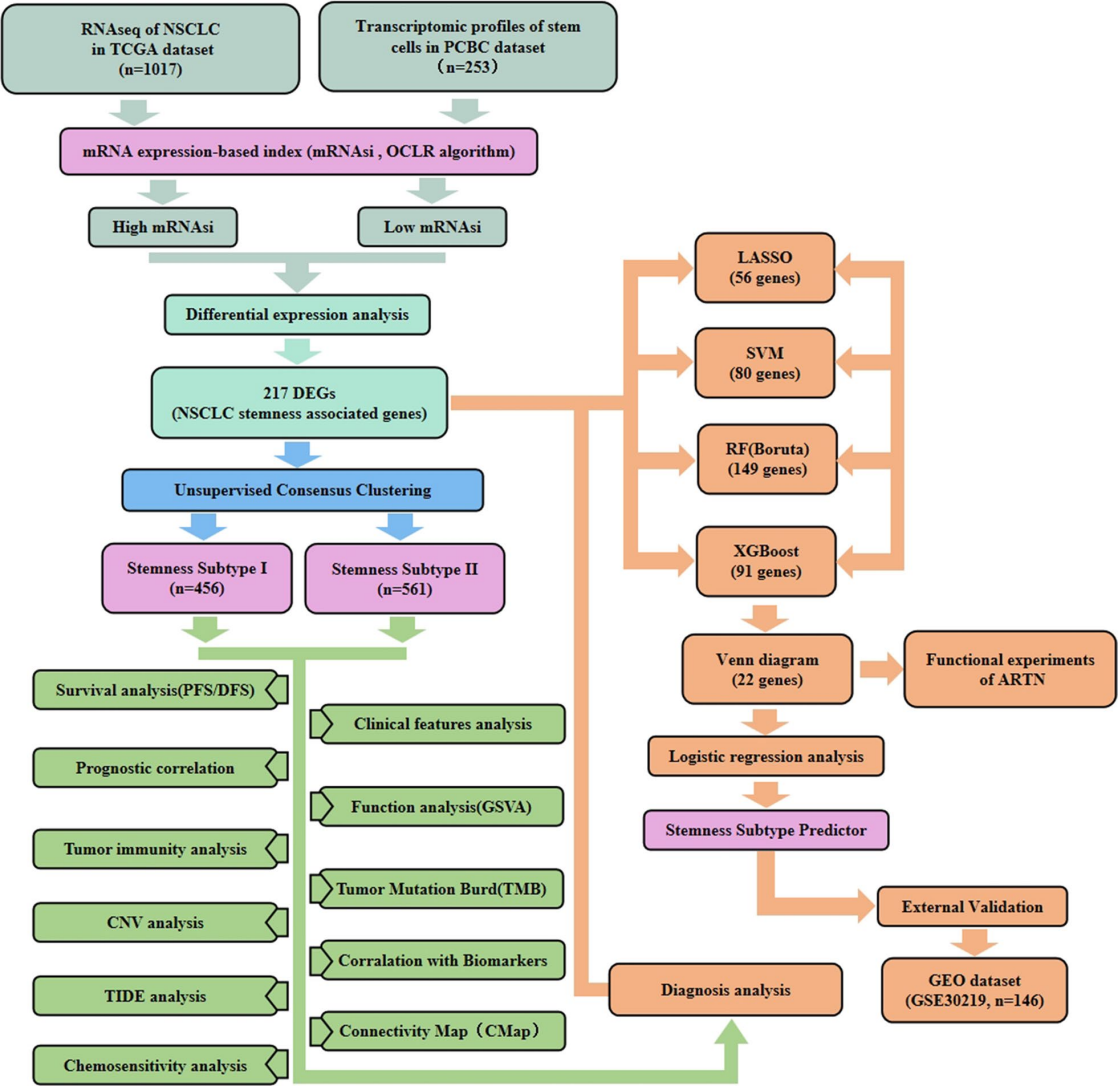
Schematic diagram containing the study design and main findings of the present study

This study was based on the TCGA-LUNG RNAseq dataset to calculate the mRNAsi of lung cancer patients. Based on the different stemness characteristics, NSCLC patients were classified into two subtypes, and the different survival outcomes, functional annotation, and clinical characteristics of both were analyzed. Then, the differences in genomic variants, tumor microenvironment, and immunogenomic patterns of patients between the two stemness subtypes were analyzed comprehensively. In addition, multiple machine learning algorithms were used to construct stemness subtype prediction models that can differentiate NSCLC patients and obtain the multivariate logistic regression equations of the characteristic genes. This is verified in the GSE30219 cohort. And cell function experiments also confirmed the ability of *ARTN*, a gene characteristic of the stemness subtype model, to promote cancer and maintain cancer cell stemness. The workflow diagram of the whole study is shown in Fig. 1. Our study aims to provide personalized survival prediction and better treatment options for NSCLC patients based on a new stemness-based molecular classification.

## Methods

### Data collection and pre-processing

The expression profile data were downloaded from the University of California Santa Cruz (UCSC Xena, https://xenabrowser.net/), including lung adenocarcinoma (515 cases) and lung squamous carcinoma (502 cases), patient clinicopathological data are displayed in Table 1, the expression matrix was log2(*X* + 1)-transformed with RSEM normalized counts. In this study, cellular data were obtained from the Progenitor Cell Biology Consortium (PCBC, https://progenitorcells.org/frontpage). In addition, the GSE30219 dataset [18] was used as external data to validate the accuracy of the model, and patient clinicopathological information is presented in Additional file 1: Table S1.

**Table 1:** Patient clinical information and pathology data

	LUAD (*N* = 515)	LUSC (*N* = 502)	Total (*N* = 1017)
*Stage*			
N/A	8 (1.6%)	5 (1.0%)	13 (1.3%)
Stage I	275 (53.4%)	244 (48.6%)	519 (51.0%)
Stage II	122 (23.7%)	162 (32.3%)	284 (27.9%)
Stage III	84 (16.3%)	84 (16.7%)	168 (16.5%)
Stage IV	26 (5.0%)	7 (1.4%)	33 (3.2%)
*Gender*			
N/A	0 (0.0%)	1 (0.2%)	1 (0.1%)
FEMALE	277 (53.8%)	130 (25.9%)	407 (40.0%)
MALE	238 (46.2%)	371 (73.9%)	609 (59.9%)
*Age*			
Mean (SD)	62.955 (15.722)	65.865 (12.670)	64.391 (14.364)
Range	0.000–88.000	0.000–90.000	0.000–90.000
*TP53*			
N/A	17 (3.3%)	21 (4.2%)	38 (3.7%)
Unaltered	228 (44.3%)	65 (12.9%)	293 (28.8%)
Altered	270 (52.4%)	416 (82.9%)	686 (67.5%)
*Mutation_Count*			
N/A	17 (3.3%)	21 (4.2%)	38 (3.7%)
< 150	218 (42.3%)	110 (21.9%)	328 (32.3%)
> 300	152 (29.5%)	123 (24.5%)	275 (27.0%)
150–300	128 (24.9%)	248 (49.4%)	376 (37.0%)
*Tobacco_Smoking_History*			
N/A	14 (2.7%)	13 (2.6%)	27 (2.7%)
1	75 (14.6%)	18 (3.6%)	93 (9.1%)
2	119 (23.1%)	133 (26.5%)	252 (24.8%)
3	135 (26.2%)	83 (16.5%)	218 (21.4%)
4	168 (32.6%)	250 (49.8%)	418 (41.1%)
5	4 (0.8%)	5 (1.0%)	9 (0.9%)

### Calculation of stemness index

Stemness index (mRNAsi) is an index calculated based on expression data describing the similarity of tumor cells to stem cells, ranging from 0 to 1, with closer to 1 indicating lower differentiation and stronger stem cell characteristics [19]. The cell expression data were trained with the OCLR (one-class logistic regression) machine learning algorithm [20], and a cell stemness model was constructed using the gelnet R packalge [21]. Based on the obtained stemness model, mRNAsi was calculated for TCGA data.

### Patient samples

Patient samples (*n* = 5) used for immunohistochemical and qRT-PCR were obtained from surgical specimens from patients of Department of Thoracic Surgery of the First Affiliated Hospital of Nanchang University, and all specimens were collected with the informed consent of the patients. Clinical information of 5 patients is shown in Additional file 1: Table S2. The specific procedure of collecting specimens was as follows: Firstly, the specimen was observed to confirm the site and scope of the tumor, and the identification with surrounding tissues and necrotic tissues was noted. The specimens were cut within 10 min after the specimens were separated from the body, and the cancer and paracancerous tissues were cut into pieces of about 1 cm in diameter and put into sterilized lyophilized tubes with numbers, which were quickly stored in liquid nitrogen tanks.

### Immunological characterization of NSCLC samples

Single sample gene set enrichment analysis (ssGSEA) was used to assess the level of immune cell infiltration in the samples. We used the complete clustering method to classify NSCLC patients into two immune subtypes (Immune Subtype I/II). Meanwhile, we used the Estimation of Stromal and Immune cells in MAlignant Tumor tissues using the Expression data(ESTIMATE) algorithm to obtain the stromal score and immune score of the two immune subtypes, which were summed to obtain the estimate score reflecting the tumor purity to assess the tumor microenvironment [22].

### High and low mRNAsi group difference analysis

“Survival” and “Survminer” R-packs were used to calculate the difference in progression-free survival (PFS) between the two groups with high and low mRNAsi, and the best cutoff values were used for grouping. The “limma” package was then used to identify differential genes (DEGs) between the high and low mRNAsi groups, using the default false discovery rate (FDR) to correct for *P* values, with |log2FC|> 2 and FDR < 0.01 being considered as significantly different genes. Next, ClusterProfiler was used for differential gene function enrichment analysis, including Gene Ontology (GO) and Kyoto Encyclopedia of Genes and Genomes (KEGG) enrichment analysis [23–25], with *q*-value < 0.05 indicating significantly enriched pathways.

### Unsupervised consensus clustering identifies stemness subtypes of NSCLC

ConsensusClusterPlus is a method that provides quantitative evidence for determining the number and membership of possible clusters in a dataset, based on the *k*-means machine learning algorithm for cluster typing of differential gene matrices using the “ConsensusClusterPlus” package in R. The clustering algorithm used was PAM to obtain stemness subtypes of NSCLC samples [26].

### GSVA analysis

The GSVA package in R was applied for KEGG enrichment analysis [27] to assess the most significantly enriched molecular pathways in the stem subtype, and KEGG gene sets were obtained from MSigDB (https://www.gsea-msgdb.org/gsea/msigdb/collection.jsp). The GSVA enrichment analysis was performed to assess the differences between the enriched molecular pathways between the high and low mRNAsi groups. We then used the limma package to analyze the difference in enrichment scores between the two groups, and |log2FC|> 0.5 and *P*.adj < 0.05 were considered molecular pathways with a significant difference.

### TIDE predicts patient response to immunotherapy

TIDE (the Tumor Immune Dysfunction and Exclusion, http://tide.dfci.harvard.edu/) was used to predict immunotherapy response in NSCLC patients and subsequently analyzed the survival difference between the two groups with high and low TIDE scores and the distribution of different cancer subtypes and also demonstrated the difference in TIDE scores among different cancer subtypes [28].

### Connectivity map (CMap) analysis

The CMap database was used to explore potential compounds associated with different stemness subtypes, which not only predicted drugs based on gene expression profiles but also revealed the mode of action (MoA) of the compounds [29]. The limma package was used to analyze differential genes between stemness subtypes, and |logFC|> 2 and FDR < 0.01 were considered genes with significant differences. The first 150 genes with positive and negative difference multiplicities, for a total of 300 different genes, were selected for querying the CMap database, and compounds with negative enrichment scores and *P* value < 0.05 were considered potential therapeutic agents for the stemness subtypes.

### Multiple machine learning methods to construct and validate stemness subtypes

The expression of DEGs was used as an input variable and the stemness subtypes were selected as the outcome and four machine learning methods were used for least absolute shrinkage and selection operation (LASSO) regression, support vector machine (SVM), Boruta (RFB), and extreme gradient boosting (XGBoost) analysis to obtain the significant genes associated with stemness typing [30–32]. The predictive performance of the four machine learning algorithms was evaluated by ROC curves to compare the area under the ROC curve (AUC). Then, common genes obtained by LASSO, SVM, RFB, and XGBoost were considered signature genes associated with stemness subtypes, and multivariate logistic regression analysis was performed on key genes to construct a stemness-based classifier.

### Validation of the impact of ARTN on NSCLC

Human NSCLC cell lines (NCIH1299, PC9, NCIH460, NCIH292) and human bronchial epithelial cells (HBE) were purchased from the Cell Bank of the Chinese Academy of Sciences (Shanghai, China). RNA was extracted from cell samples and patient samples using TRIzol RNA Extraction Reagent (Invitrogen™, 15596026) according to the instructions. After removal of residual gDNA, reverse transcription reactions were performed using 1ug of total RNA as template. This was followed by real-time quantitative PCR(qPCR) using 2 × qPCR MasterMix(KAPA, KM4103) on a LightCycler 480 instrument (Roche). Relative gene expression was analyzed by the 2-ΔΔCt method, using Gapdh as an internal control. qPCR and in vitro cancer cell function assays were also performed as described previously [33, 34]. qPCR primer sequences are shown in Additional file 1: Table S3. siRNA sequences of *ARTN* were purchased from GenePharma Co. The sequences are listed in Additional file 1: Table S4. In addition, immunohistochemical assays were used to verify the difference in protein expression of ARTN in normal lung tissue and lung cancer tissue.

### Immunohistochemistry

Tissue slides were prepared from patient samples, and immunohistochemical staining was performed with anti-ARTN antibodies to assess ARTN expression in the tissue. The procedure was as follows: The tissue slides were deparaffinized, rehydrated, and subjected to antigen repair. Subsequently, endogenous peroxidase activity was blocked with 6% hydrogen peroxide. Slides were washed three times with PBS and then incubated overnight at 4 °C with anti-ARTN antibody. After three washes with PBS, the slides were incubated with horseradish peroxidase-conjugated secondary antibody for 1 h. The nuclei were stained with hematoxylin solution. Anti-ARTN antibody was purchased from ZENBIO (R23532).

### Statistics analysis

Wilcoxon rank-sum test was used to compare differences between two groups, and the Kruskal–Wallis test was used to compare multiple groups. Correlation between normally distributed variables was tested by the Pearson correlation test, and the correlation between non-normally distributed variables was tested by the Spearman correlation test. For correlation analysis of categorical variables, the chi-square test was used. *P* value < 0.05 and correlation coefficient *R* > 0.3 were considered to be significantly correlated. Statistical analysis for this study was performed using SPSS 22.0 and R 4.1.0 software.

## Results

### Correlation of stemness index with clinical characteristics and gene mutations

**Fig. 2:**
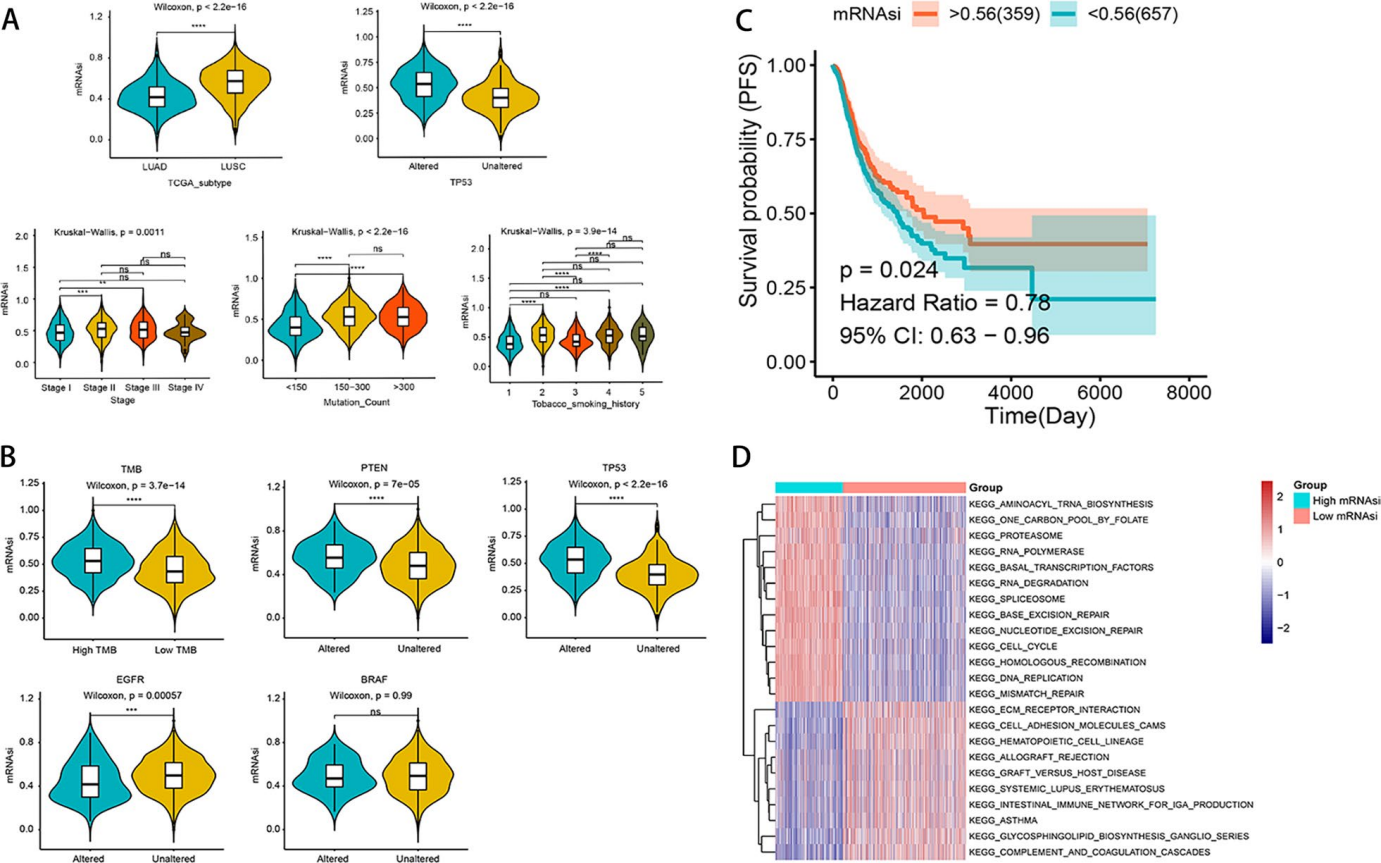
Clinical and molecular characteristics of NSCLC patients associated with stemness index (mRNAsi). **A** Violin plot showing mRNAsi differences in different clinical feature groupings. **B** Relationship between mRNAsi and different feature groupings in NSCLC patient samples grouped by TMB height, and *PTEN, TP53, EGFR, BRAF* mutation status. *Indicates *P* value < 0.05,**indicates *P* value < 0.01, ***indicates *P* value < 0.001, ****indicates *P* value < 0.0001. **C** PFS survival analysis, red line represents high mRNAsi group, green line represents low mRNAsi group. **D** Differential enrichment pathways between the two groups of high and low mRNAsi are shown, each small box represents each patient’s enrichment score, with color changes indicating high or low scores: red for high scores and blue for low scores. The grouping of each patient is shown at the top of the heat map

Using the OCLR algorithm, we calculated the mRNAsi for each patient. Subsequently, we evaluated the relationship between sample mRNAsi and clinicopathological features, grouped according to different clinical features, and the results showed that the subgroups with LUSC, Stage II, and Stage III, Mutation Count of 150–300 and > 300 mRNAsi were significantly higher (Fig. 2A and Additional file 2: Fig. S1A). The results showed that mRNAsi was higher in high TMB and low TMB according to the median TMB, and mRNAsi was significantly higher in *PTEN, TP53* mutated group than in an unmutated group, while mRNAsi was higher in *EGFR* unmutated group than in mutated group (Fig. 2B & Additional file 2: Fig. S1B). PFS was higher in the high mRNAsi group than in the low mRNAsi group (Fig. 2C), and there were significant differences in clinical characteristics between the two groups, including smoking history, stage, mutation counts, *TP53* mutation status, TCGA subtype (Additional file 2: Fig. S1C).

### Functional annotation of high and low mRNAsi groups and mutation of DEGs

A total of 217 genes (DEGs) with significant differences between the high mRNAsi and low mRNAsi groups were identified (Additional file 2: Fig. S1D and Additional file 1: Table S5). GO and KEGG enrichment analysis of these differential genes demonstrated the top 30 significantly significant BPs, CCs, and MFs (Additional file 3: Fig. S2A), and 6 significantly enriched pathways (Additional file 3: Fig. S2B). In addition, we analyzed the differences between the enriched molecular pathways between the high and low mRNAsi groups using GSVA, and the results showed that the high mRNAsi group was mainly related to DNA damage repair, homologous recombination, etc., while the low mRNAsi group was related to asthma, complement system, etc. (Fig. 2D). To understand the mutation profile of DEGs, we showed the 10 genes with the highest mutation frequency in DEGs, namely *CNTNAP2, MUC5B, ADAMTSI6, DMBT1, ITGA8, C6, ROS1, SCN7A, CACNAIB, FAM83B,* which were mutated in 51.82% of the samples and the mutation type was mostly missense mutation (Additional file 3: Fig. S2C). In addition, we analyzed the CNV of 217 DEGs, and the results showed that there were 100 genes with significant amplification and 95 genes with significant deletion (Additional file 3: Fig. S2D).

### Identification of two stemness subtypes with different survival outcomes, functional annotation and clinical features

The consistency clustering method was used to typify the sample based on the expression of 217 DEGs, and the consistency matrix plot had the highest consistency when *K* = 2 (Additional file 4: Fig. S3A) and the lowest CDF compared to other clusters (Additional file 4: Fig. S3B), and the delta area score was highest at *K* = 2 (Additional file 4: Fig. S3C). Therefore, patients were distinguished into two subtypes, namely Stemnness Subtype I (456 cases) and Stemness Subtype II (561 cases), and the expression of DEGs between stemness subtypes is shown in Additional file 4: Fig. S3D.

**Fig. 3:**
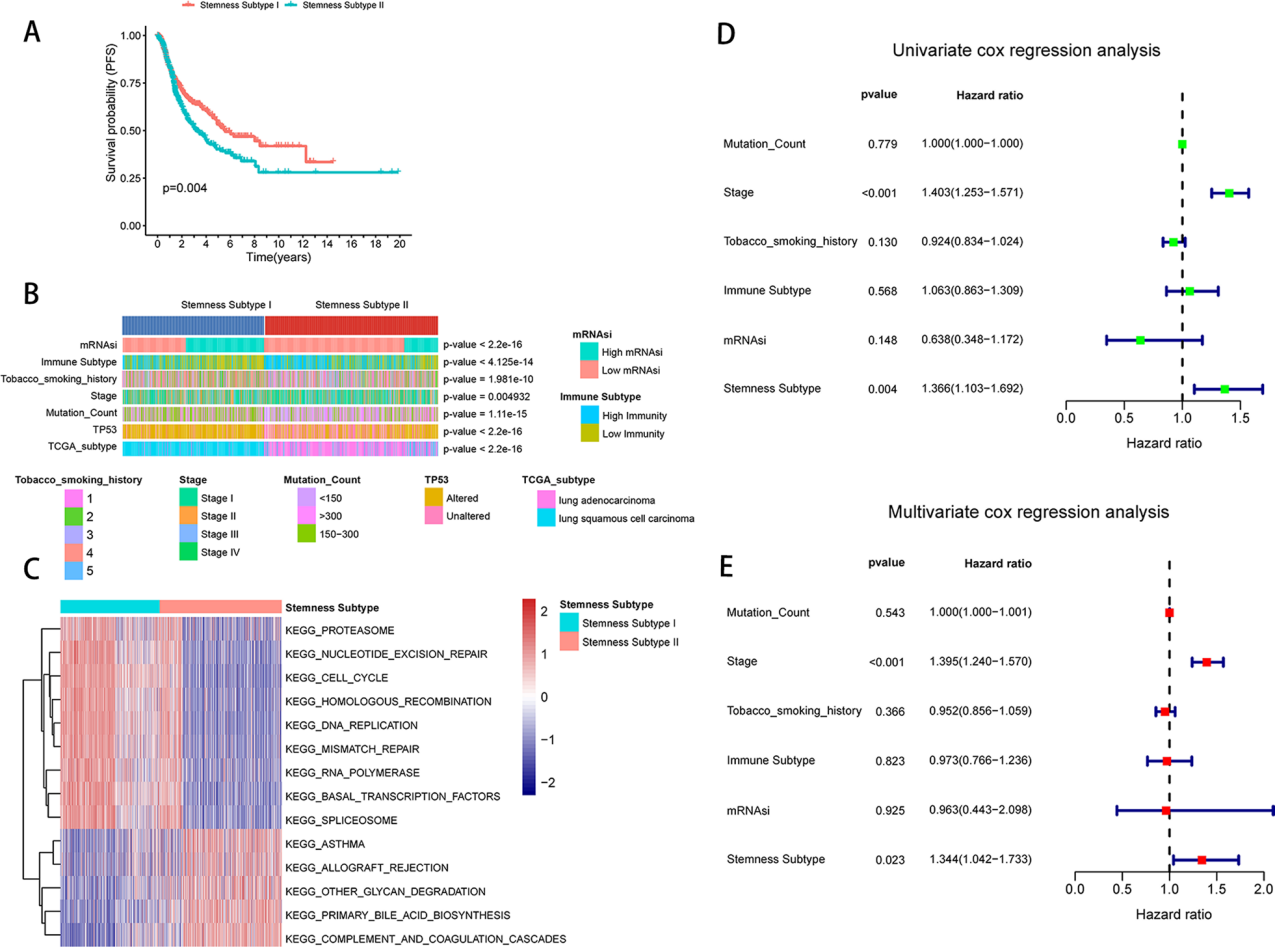
Stemness subtypes are associated with clinical characteristics of NSCLC patients and can act as independent predictors. **A** Survival analysis showing the difference in survival between Stemness Subtype I and Stemness Subtype II. **B** Differences in clinical characteristics between the Stemness Subtype I and Stemness Subtype II groups. Columns indicate samples and rows indicate clinical characteristics. **C** Display of differential enrichment pathways between Stemness Subtype I and Stemness Subtype two groups. Each small box represents the enrichment score of each patient, and the color change indicates the high or low enrichment score: red indicates high score, blue indicates low score, and the subgroup of each patient is shown at the top of the heat map. **D** Univariate Cox regression analysis of the TCGA dataset with meaningful factors Stage and Stamness Subtype. Multivariate Cox regression analysis of the TCGA dataset with meaningful factors Stage and Stamness Subtype

Further, the survival differences between subtypes were analyzed (Fig. 3A), and the results showed that patients in the Stemness Subtype I group had a higher PFS than Stemness Subtype II. In addition, there were significant differences in clinical characteristics between the Stemness Subtype I and Stemness Subtype II(*P* < 0.05), including mRNAsi grouping, immune subtype, tobacco smoking history, stage, TP53 mutation status, and TCGA subtype (Fig. 3B). GSVA analysis of Stemness Subtype I and Stemness Subtype I groups showed a total of 14 pathways with significant differences (|log2FC|> 0.5, *P*.adj < 0.05), and the results showed that Stemness Subtype I was mainly related to DNA damage repair and homologous recombination, while Stemness Subtype II was associated with primary bile acid synthesis, asthma, etc. (Fig. 3C). To assess whether the stemness subtype could independently predict prognosis, we included mutation count, stage, tobacco smoking history, immune subtype, mRNAsi, and stemness subtype for univariate and multivariate cox regression analyses, and the results showed that the stemness subtype was associated with primary bile acid synthesis and asthma. The results showed that the prognostic analysis of stemness subtype was significant in both single factor (*P* = 0.004, HR = 1.366, 95% CI 1.103–1.692) and multifactor (*P* = 0.023, HR = 1.344, 95% CI 1.042–1.733) (Fig. 3D & Fig. 3E), indicating that stemness subtype can act as an independent prognostic factor.

The expression of DEGs in the validation set (GSE30219) was classified using a consensus clustering approach to the samples, and the cluster analysis of the samples identified two subtypes, defined as Stemnness Subtype I (57 cases) and Stemness Subtype II (89 cases). Further survival analysis showed that Stemness Subtype II had a higher disease-free survival (DFS) than Stemness Subtype I (Additional file 5: Fig. S4).

### Differences in TMB, genetic mutations, and immune characteristics among stemness subtypes

**Fig. 4:**
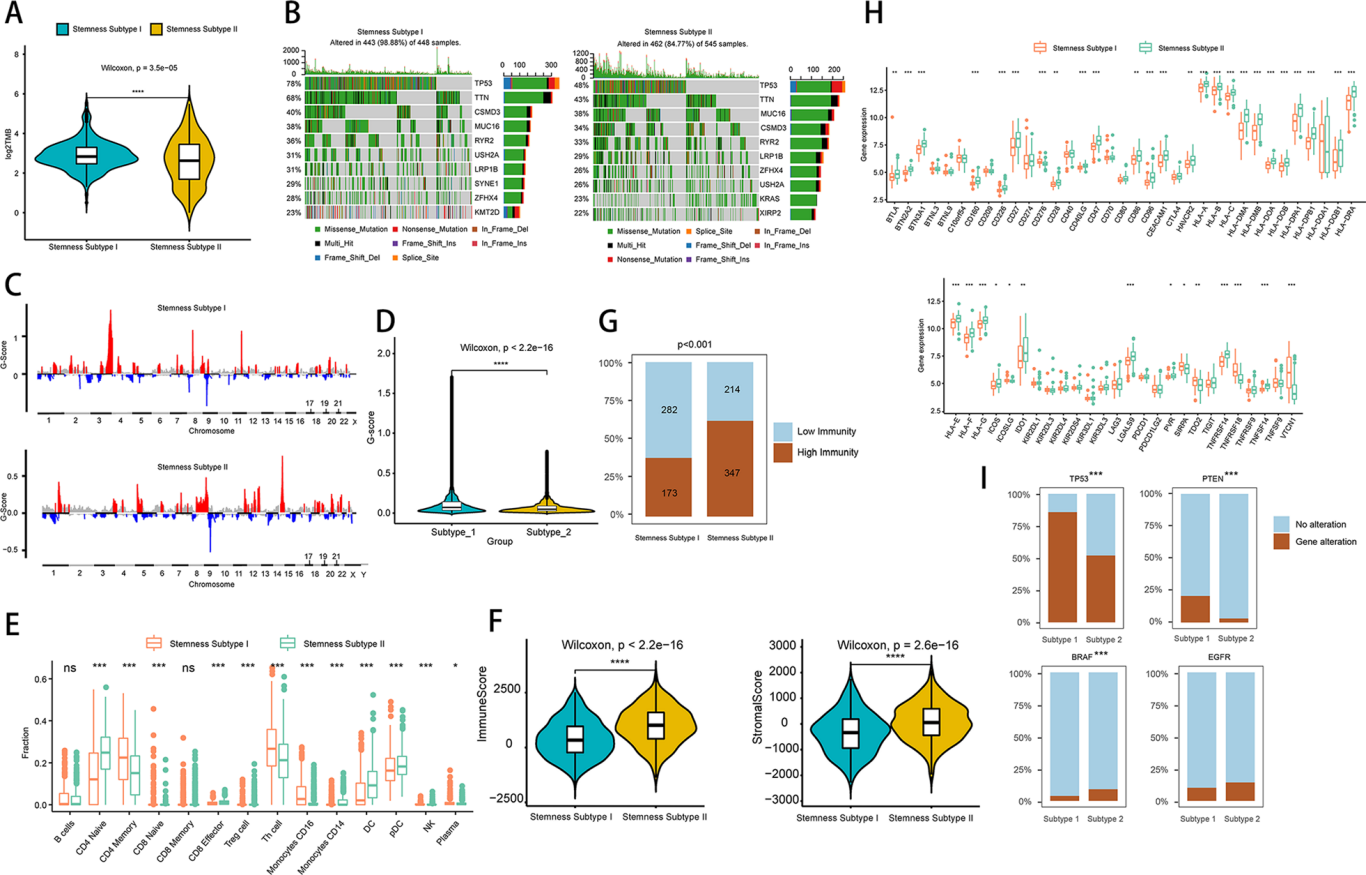
Functional analysis of stemness subtypes and immunological analysis. **A** Violin diagram showing TMB differences between stemness subtypes, green indicates Stemness Subtype I, yellow indicates Stemness Subtype II. **B** Waterfall diagram showing the 10 genes with the highest mutation frequencies in Stemness Subtype I and Stemness Subtype II samples, different colors indicate different mutation types. The different colors indicate the different mutation types. **C** Copy number difference analysis of Stemness Subtype I and Stemness Subtype II, red indicates amplification and blue indicates deletion. gistic analysis assigns a G-score to each mutation, which indicates the magnitude of the mutation. **D** G-score differences between Stemness Subtype I and Stemness Subtype II were analyzed using box plots. **E** Differences in immune cells in different subgroups. **F** Comparison of differences in immune scores, stromal scores in different stemness subtypes by violin plots, ****Indicates *P* value < 0.0001. **G** Differences in immune subtype distribution in different stemness subtypes, sky blue indicates low immunity, brown indicates high immunity. **H** Analysis of differences in immune checkpoint genes. Red indicates Stemness Subtype I, green indicates Stemness Subtype II. **I** Gene mutation in different stemness subtypes, sky blue indicates no mutation in the sample, brown indicates mutation in the sample, * indicates *P* value < 0.05, ** indicates *P* value < 0.01, *** indicates *P* value < 0.001, ns indicates no statistical significance

TMB was higher in Stemness Subtype I than in Stemness Subtype II (Fig. 4A), and *TP53, TTN, CSMD3, MUC16, RYR2, USH2A, LRPIB, SYNEKZFHX4,* and *KMT2D* were mutated in 98.88% of the samples in Stemness Subtype I, while the genes with higher mutation frequency in Stemness Subtype II were *TP53*, *TTN*, *MUC16*, *CSMD3*, *RYR2*, *LRP1B*, *ZFHX4*, *USH2A*, *KRAS*, *XIRP2*, 84.77% of the samples were mutated ( Fig. 4B). To understand the CNV differences among different stemness subtypes, we performed the analysis using the GISTIC module in genepatern and visualized the data obtained from the analysis using maftools (Fig. 4C). In addition, the G-score of Stemness Subtype I was higher than that of Stemness Subtype II (Fig. 4D). In addition, we analyzed the mutations of biomarkers among different stemness subtypes, and the results showed that the number of TP53 and PTEN mutations was higher in Stemness Subtype I, and BRAF was more mutated in Stemness Subtype II.

Immunoreactivity analysis between stem subtypes showed the Overall Score in Stemness Subtype II than in Stemness Subtype I, suggesting that Stemness Subtype II is less likely to benefit from immune checkpoint therapy. The results of immune cell infiltration showed differences in the proportion of immune cell infiltration between Stemness Subtype I and Stemness Subtype II, including CD4 + T cell, CD8 + T cell, CD4 memory cell, etc. (Fig. 4E). The results of the immune microenvironment analysis showed that both immune score and stromal score were higher in Stemness Subtype II than in Stemness Subtype I (Fig. 4F). In addition, we analyzed the immune subtype differences between different stemness subtypes in combination with immune subtypes, and the results showed that there were more samples with high immunity in Stemness Subtype II and more low immunity in Stemness Subtype I (Fig. 4G). In Fifty-four immune detection sites with differential gene expression, the analysis showed that the expression of *TP53*, *PTEN*, and *BRAF* was significantly different among the stemness subtypes (Fig. 4H, I).

### TIDE predicts immunotherapy response between cancer types

**Fig. 5:**
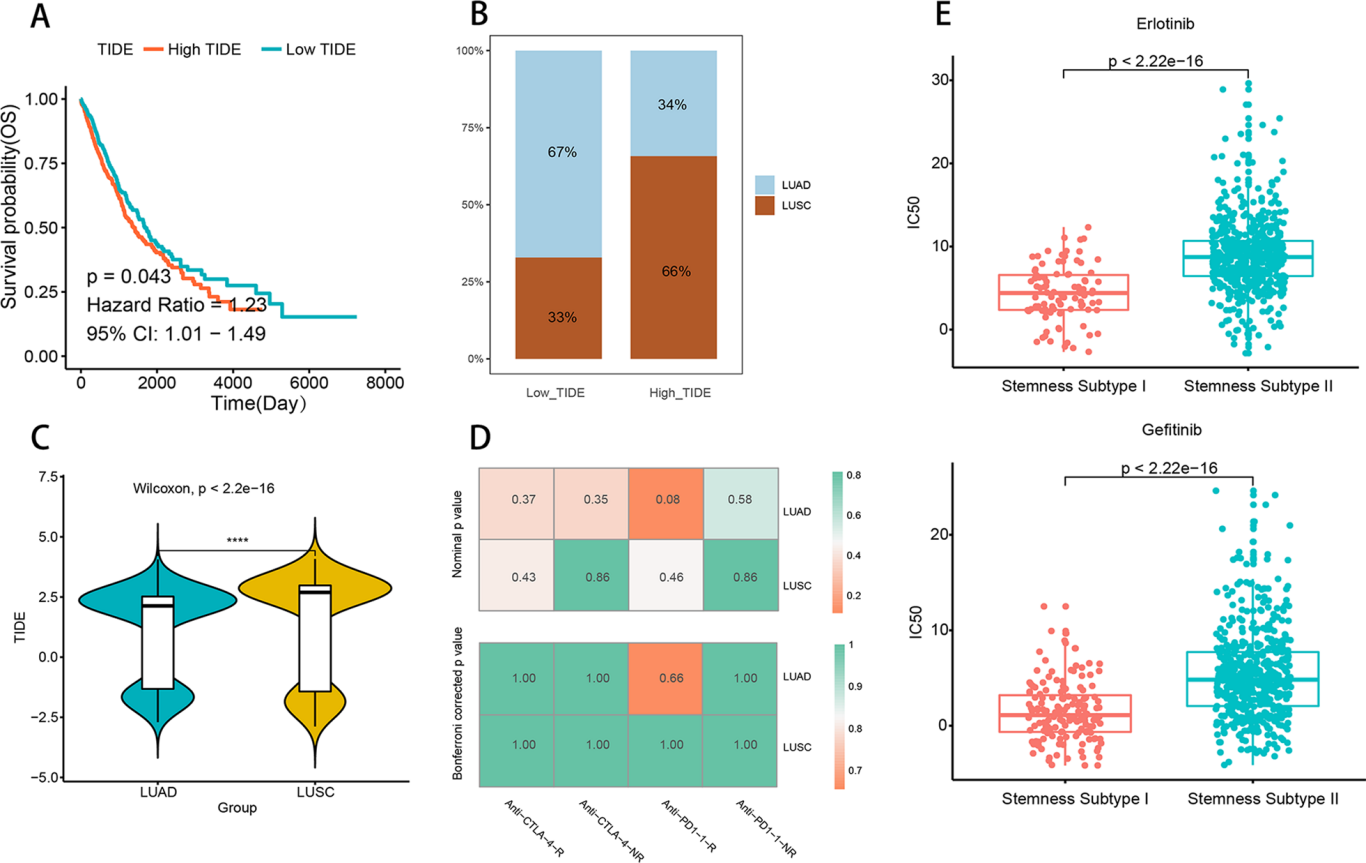
TIDE predicts immunotherapy response between cancer types. **A** Survival difference between the two groups of high and low TIDE, green indicates low TIDE score, red indicates high TIDE score. **B** Distribution of different cancer subtypes between two groups of high and low TIDE, sky blue indicates LUAD, brown indicates LUSC. **C** TIDE difference between cancer subtypes, green indicates LUAD, yellow indicates LUSC, ****indicates *P* value < 0.0001. **D** Submap analysis demonstrating the predicted efficacy of different cancer subtypes in immunotherapy

TIDE scores were obtained by online analysis of gene expression matrices, and the results of survival analysis showed that high TIDE had a lower survival rate according to TIDE scores in two groups (Fig. 5A). In addition, LUAD accounted for 67% in the low TIDE group and 66% in the High TIDE group with LUSC (Fig. 5B), and TIDE scores differed across cancers (*P* < 0.0001) and were higher in LUSC (Fig. 5C). To analyze the efficacy of anti-*PD-1* and anti-*CTLA4* treatments in different cancer subtypes, we extracted data on immunotherapy in melanoma patients as a reference, and 47 immunotherapy patients were included in the analysis. We predicted the NSCLC data using submap in genepatern to obtain the efficacy of anti-*PD1* and anti-*CTLA4* treatment, but the results showed no significant treatment (Fig. 5D).

### Drug sensitivity analysis and identification of potential compounds between stemness subtypes

To understand the sensitivity of different stemness subtypes to antineoplastic agents, we analyzed the sensitivity of different stemness subtypes to two antineoplastic agents, erlotinib and gefitinib. The results showed a difference in the sensitivity of Stemness Subtype I and Stemness Subtype II to erlotinib and gefitinib, with Stemness Subtype I having a higher sensitivity (Additional file 6: Fig. S5A).

CMap helps to explore potential compounds between stemness subtypes of NSCLC, and of the 771 differential genes between Stemness Subtype I and Stemness Subtype II, the top 150 genes with positive and negative difference multiplicities were included in the CMap database query. Compounds with negative enrichment scores and *P* < 0.05 were selected for display and analyzed for MoA of each compound, showing that 30 molecular pathways were being targeted by 47 compounds in Stemness Subtype I; while 29 molecular pathways were targeted by compounds in Stemness Subtype II (Additional file 6: Fig. S5B).

### Machine learning for constructing stemness subtype models and model validation

**Fig. 6:**
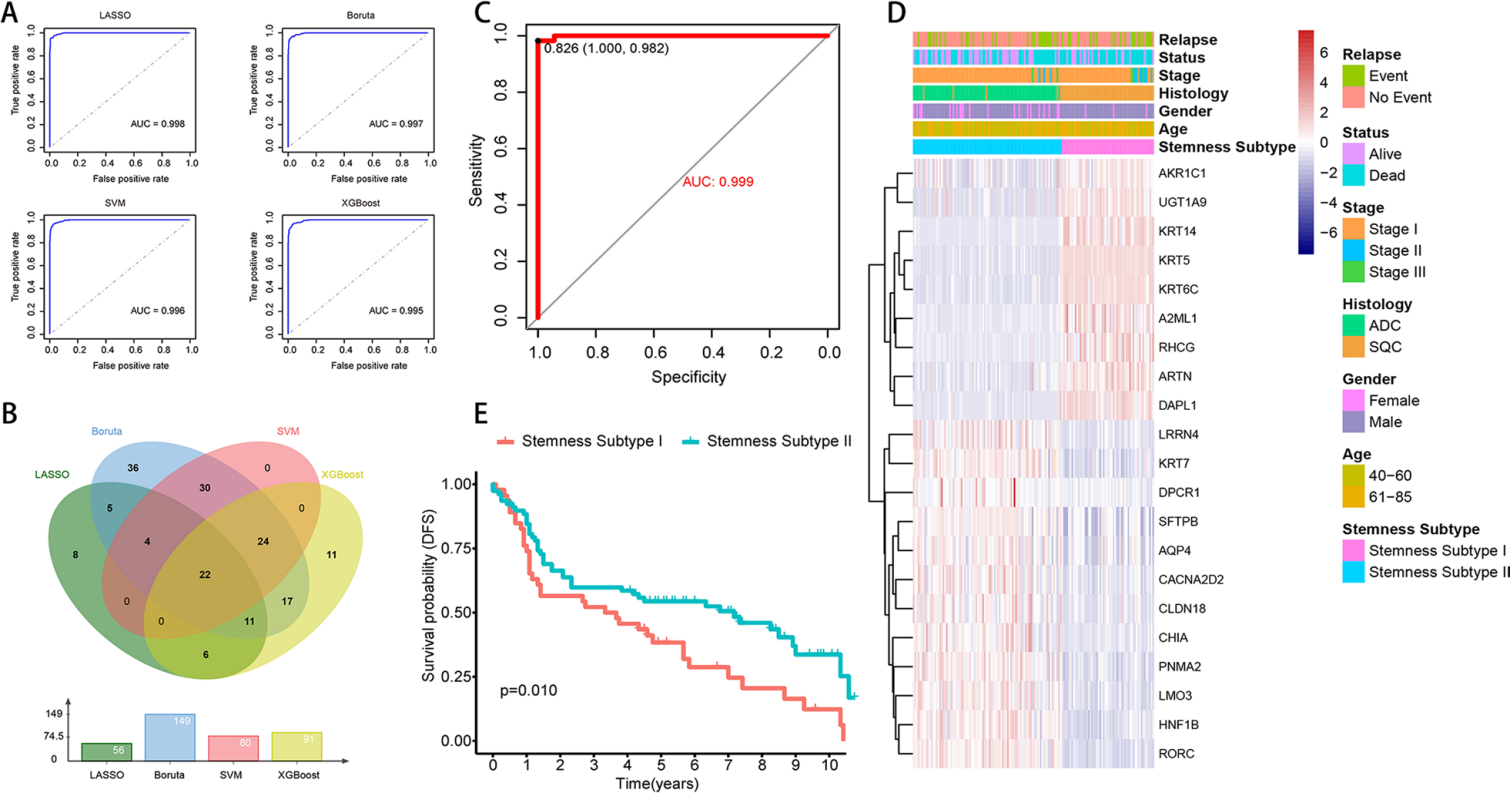
Machine learning to construct stemness models. **A** LASSO, Boruta, SVM and XGBoost feature selection performance evaluation, AUC is generated by ROC curve analysis. **B** The feature genes shared by the four machine learning algorithms were identified by VENN plots, totaling 22 important feature genes. **C** ROC curves of 22 gene signatures predicted to validate stemness subtypes.** D** Heat map showing the expression of the signature genes in the validation set, with red indicating high expression and blue indicating low expression. The top of the heat map shows the distribution of clinical features for each patient, including Stemness Subtype, Age, Gender, Histology, Stage, Stage, Status, Relapse. **E** Survival differences between Stemness Subtype I and Stemness Subtype II groups. Red indicates Stemness Subtype I and green indicates Stemness Subtype II

Four machine learning algorithms (LASSO, Boruta, SVM, and XGBoost) were used to analyze the expression matrices based on 217 DEGs, respectively, and finally to obtain the important signature genes associated with the stemness subtypes. the ROC analysis showed that the four machine learning algorithms could perform the signature gene selection well (AUCs > 0.95, Fig. 6A). The LASSO, Boruta, SVM, and XGBoost analyses yielded 56, 149, 80, and 91 significant feature genes, respectively, with 22 intersecting genes (Fig. 6B). After that, multivariant logistic regression analysis was performed and prediction models were constructed. The formula to obtain the predicted stemness subtypes was as follows: stemness subtype prediction score = 4.595 + 0.00065 × (*ARTN* expression) − 0.363 × (*LRRN4* expression) + 0.125 × (*KRT14* expression) − 0.326 × (*DPCR1* expression) − 0.12 × (*SFTPB* expression) + 0.065 × (*DAPLI* expression) − 0.037 × (*PNMA2* expression) + 0.108 × (*A2ML1* expression) + 0.47 × (*AKRICI* expression) − 0.207 × (*CACNA2D2* expression) − 0.2879 × (*LMO3* expression) + 0.366 × (*KRT5* expression) − 0.167 × (*CHIA* expression) + 0.28 × (*KRT6C* expression) − 0.27 × (*KRT7* expression) − 0.1986 × (*HNFIB* expression) − 0.216 × (*RORC* expression) + 0.049 × (*UGTIA9* expression) − 0.036 × (*RHCG* expression) − 0.125 × (*AQP4* expression) − 0.155 × (*CLDN18* expression) − 0.1799 × (*MUC21* expression).

We predicted the validation set, and the samples with prediction score < 0.826 were Stemness Subtype II (90 cases), and those with prediction score > 0.826 were Steminess Subtype I (56 cases). The ROC curves showed that the differentiation between Stemness Subtype I and Stemness Subtype II with the AUC was 0.999 (Fig. 6C), and the expression distribution of the 22 important characteristic genes is shown in Fig. 6D. Survival analysis showed that Stemness Subtype II had a higher DFS than Stemness Subtype I (Fig. 6E). We further analyzed the differences in the distribution of clinical characteristics of the stemness subtypes in the validation set, and the results showed differences in status, stage, histology, and gender between the Stemness Subtype I and Stemness Subtype II groups (Additional file 7: Fig. S6A). Tumor immune infiltration analysis showed partial differences in the ratio of immune cells between Stemness Subtype I and Stemness Subtype II (Additional file 7: Fig. S6B). Tumor microenvironment analysis showed that immune score and stromal score were higher in Stemness Subtype II and tumor purity was higher in Stemness Subtype I (Additional file 7: Fig. S6C). We analyzed the differences in gene expression of 54 immune checkpoints between the validated set of stemness subtypes, and the results showed that Stemness Subtype I and Stemness Subtype II had significant differences in multiple immune checkpoint genes. We analyzed the differences in gene expression of 54 immune checkpoints between the validation set of stemness subtypes and showed that Stemness Subtype I and Stemness Subtype II had significant differences in multiple immune checkpoint genes (Additional file 7: Fig. S6D). In addition, TIDE scores were obtained by online analysis using the gene expression volume matrix, and the survival differences between the high and low TIDE groups were analyzed according to the TIDE scores, and the results showed that the high TIDE group had lower DFS (Additional file 7: Fig. S6E). Further analysis of the distribution of different cancer subtypes between the high and low TIDE groups showed that ADC accounted for 67% in low TIDE, 66% in SQC in high TIDE (Additional file 7: Fig. S6F), and TIDE was higher in SQC in different cancer types (Additional file 7: Fig. S6G). Drug sensitivity analysis showed that Stemness Subtype I was more sensitive to erlotinib and gefitinib (Additional file 7: Fig. S6H).

### Functional validation of *ARTN* in NSCLC

**Fig. 7:**
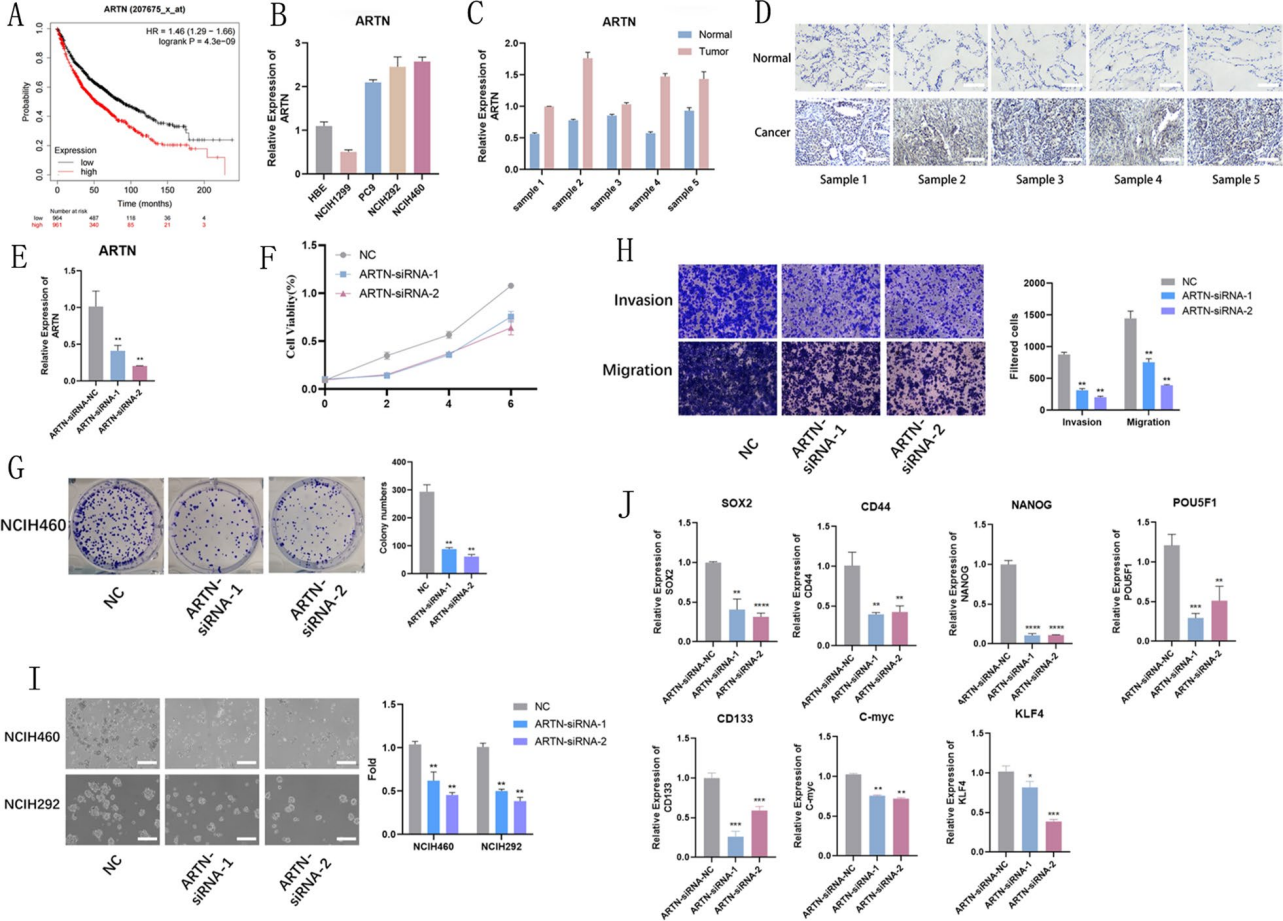
Interference with *ARTN* inhibits the proliferation, migration, invasion and stemness of lung cancer cells. **A** K–M analysis shows that patients with high *ARTN* expression have a worse prognosis.** B** qRT-PCR results demonstrate the difference in transcript levels of *ARTN* in normal lung epithelial cells (HBE) and lung cancer cells (NCIH1299, PC9, NCIH292, NCIH460). **C** qRT-PCR results demonstrating the differences in transcript levels of *ARTN* in clinically paired tissue samples. **D** Differential protein expression of ARTN in clinical paired tissue samples detected by immunohistochemistry. **E** qRT-PCR to detect the interference effect of *ARTN*-siRNAs. **F** Lung cancer cell growth after *ARTN*-siRNAs interference. (**G**) Clonal colony formation after *ARTN*-siRNAs interference. **H** Invasion and migration of lung cancer cells after *ARTN*-siRNAs interference. **I** Stem cell formation in lung cancer cells after *ARTN*-siRNAs interference. **J** Changes in transcript levels of stem Markers (*SOX2, CD44, NANOG, POU5F1, CD133, C-myc, KLF4*) after *ARTN*-siRNAs interference

We performed bioinformatics analysis and functional validation of 22 signature genes, among which *ARTN* exhibited relatively significant oncogenic ability. Kaplan–Meier plotter analysis showed that lung cancer patients with high *ARTN* expression had poor survival (Fig. 7A). To further explore the biological function of ARTN in non-small cell lung cancer, qRT-PCR was used to detect differences in ARTN expression in normal lung epithelial cells(HBE) and lung cancer cells(NCIH1299, PC9, NCIH292, NCIH460) (Fig. 7B), and differential expression of ARTN in clinical paired tissue samples was also detected by qRT-PCR(Fig. 7C) and immunohistochemical staining(Fig. 7D). We performed a series of cellular functional assays after interfering with ARTN expression(Fig. 7E). Cell growth assay and clonal colony formation assay showed that interference with *ARTN* inhibited cell proliferation (Fig. 7F, G). Transwell assay showed that interference with *ARTN* expression inhibited the invasion and migration ability of lung cancer cells (Fig. 7H). In addition, to investigate the effect of *ARTN* on the maintenance of stemness of lung cancer cells, we performed a stemness sphere-forming assay (Fig. 7I) and examined the difference in the expression of cancer stem cell markers (*SOX2*, *CD44*, *NANOG, POU5F1*, *CD133, C-myc, KLF4*), and the transcript levels of cancer stem cell markers were significantly reduced (Fig. 7J).

## Discussion

In this study, we conducted an in-depth analysis of the relationship between stemness characteristics of NSCLC and tumor immune microenvironment and treatment outcome, constructed a model to differentiate subtypes based on stemness characteristics of lung cancer patients, and performed external validation. First, we used the OCLR algorithm to calculate the mRNAsi of 1017 NSCLC patients. Then, the tumor microenvironment, tumor purity, and the abundance of stromal and immune cells in NSCLC patients were assessed using ESTIMATE. After analyzing the interaction between lung cancer stemness characteristics and tumor immune microenvironment, CIBERSORT also analyzed immune cell infiltration. Unsupervised consistent clustering of DEGs between high and low mRNAsi groups was performed, and NSCLC patients were classified into two stemness subtypes to elucidate the correlation between stemness subtypes and clinical features by comparing the differences in clinical features. Subsequently, we found that Stemness Subtype I was more sensitive to erlotinib and gefitinib chemotherapy; And that patients in this group had better DFS and worse PFS. We identified potential compounds targeting stemness-related genes by CMap analysis, which laid the foundation for the study of lung cancer treatment. In addition, multivariant logistic regression equations for 22 signature genes were obtained by applying LASSO, SVM, RFB, and XGBoost machine learning methods and validated them in the GEO non-small cell lung cancer dataset cohort. Cell function experiments demonstrate the ability of the signature gene ARTN to promote cancer and maintain the stemness of lung cancer cells.

In recent years, lung cancer stem cells have gradually become a new direction in lung cancer treatment. Although there are no clear criteria for the identification of markers of lung cancer stem cells, there exist some cell surface proteins or enzymes that are widely expressed in lung cancer stem cells and so on that are widely recognized as positive markers, such as *CD133*, *CD166*, *ALDH*, *ABCG2*, *CD44* and *CD166* [35–37]. The mechanisms of targeting drugs against these stem cell-positive markers are also being explored experimentally. Lysergic acid (*SDA*) is known to downregulate tumor cell sphere-forming ability by regulating *ABCG2* expression [38]. Trifluoperazine treatment of lung cancer stem cells reduced the sphere-forming ability of lung cancer cells and down-regulated *CD44* and *CD133* expression, and trifluoperazine in combination with gefitinib or cisplatin effectively controlled drug resistance in lung cancer cells [39]. It was concluded that mRNAsi could help to elucidate the pathways by which tumors undergo dedifferentiation and identify novel targets for anticancer drugs, thus helping to develop novel therapies to inhibit further tumor progression [40]. In this study, we calculated the mRNAsi of 1017 NSCLC patients and further analyzed the clinical characteristics correlation, prognostic correlation, and immune correlation of mRNAsi. Interestingly, based on the results of our analysis, we found that, unlike most tumors, the high mRNAsi group had a better prognosis in non-small cell lung cancer, a result consistent with the higher mRNAsi of Stemness Subtype I having a better prognosis, the reason for which may be related to the high drug sensitivity of this group.

We defined 22 signature genes identified by four machine learning methods as stemness subtype predictors. Numerous studies have shown that these stemness subtype predictors are closely associated with tumor development and maintenance of tumor cell stemness, such as cytokeratin 14 (*KRT14*), a marker of bladder stem cells, which plays an important role in bladder tumorigenesis [41]; animal experiments revealed that *CLDN18* is highly expressed in the alveolar epithelium and knockdown of *CLDN18* leads to lung enlargement, parenchymal expansion, alveolar epithelial type II (*AT2*) cell abundance and regulates lung stem and progenitor cell homeostasis and tumorigenesis by mediating Yes-associated protein (*YAP*) activation [42]. Artemin (*ARTN*) has been reported to be able to promote human non-small cell lung cancer (NSCLC) progression. Overexpression of *ARTN* stimulates survival, migration, and invasion of NSCLC cell lines; overexpression of *ARTN* in H1299 cells (p53 deficient) leads to the formation of larger tumors that are highly proliferative, aggressive, and metastatic. Increased *ARTN* expression in NSCLC also predicts metastasis to lymph nodes and increased grading of certain NSCLC subtypes. Mechanistic studies suggest that *ARTN* increases *BCL2* expression through transcriptional upregulation and that inhibition of *BCL2* eliminates the oncogenic properties of *ARTN* in NSCLC cells. Consistently, both siRNA interference and functional inhibition of endogenous *ARTN* with antibodies reduced the oncogenicity and invasiveness of NSCLC cells [43]. However, *ARTN* has little to no reported effect on maintaining lung cancer cell stemness. In this study, we used siRNA to interfere with *ARTN* expression in lung cancer cells to investigate the effect of *ARTN* on non-small cell lung cancer. The results showed that tumor cell proliferation, migration ability, and stemness sphere-forming ability were inhibited after siRNA interference. qRT-PCR showed that the transcript levels of stemness markers (*SOX2, CD44, NANOG, POU5F1, CD133, C-myc, KLF4*) were significantly reduced. These results suggest that ARTN is a non-small cell lung cancer procancer factor and plays an important role in maintaining stemness.

Immunotherapy has entered the clinic as a first-line treatment for NSCLC, mainly including immune checkpoint inhibitors, antitumor vaccines, and cellular immunotherapy [44]. However, the role of immunotherapy in controlling metastasis and recurrence has not been affirmed and further studies are still needed. In addition, the effectiveness of immunotherapy for NSCLC remains limited, such as the low sensitivity and specificity for *PD-L1* expression, and although *PD-1/PD-L1* immune checkpoint inhibitors have become the new standard of care for advanced NSCLC, immunotherapy alone has a high proportion of patients with primary nonresponse [45]. In addition, immunotherapy suffers from increased drug resistance, frequent and uncontrollable adverse effects, and unclear prevention of metastasis, which hinder its further development. In this study, a new classification of NSCLC based on tumor stemness was proposed. The results showed that patients with poorer prognosis among the stemness subtypes had lower immune scores and stromal scores, higher anticancer immunity activity scores (overall score), and reduced expression of immunosuppressive checkpoints, suggesting a poorer response to immune checkpoint inhibitors. These results are of clinical value for follow-up studies and may provide a new idea for screening patients with predictive immune status and sensitivity to immunotherapy in lung cancer patients.

However, there are some limitations to this study. Because the number of patients currently receiving immunotherapy is very limited, and because the results of this study are based on transcriptomic data analysis from public databases, the relationship between stem cell subtypes and immunotherapy responsiveness needs to be validated in future immunotherapy cohorts. In conclusion, immunotherapy still has great potential in NSCLC, and screening patients who may benefit from immunotherapy is one of the important tasks at present.

## Conclusion

In summary, this study provides additional evidence that cancer stem cells play a key role in the tumor immune microenvironment of NSCLC patients. Based on the stemness characteristics of NSCLC patients, we constructed a novel subtype classifier, which can not only provide a reference for basic research before the development of lung cancer stem cells and lung carcinogenesis but also be applied as a potential screening method for NSCLC patients in clinical treatment.

### Supplementary Information

**Additional file 1**. **Table S1**: Patient clinical information of GSE30219. **Table S2**: Clinical information of 5 NSCLC patients. **Table S3**: Primers of Quantitative Real-time PCR. **Table S4**: The siRNA specific for ARTN mRNA. **Table S5**: 217 DEGs between high and low mRNAsi group.

**Additional file 2**. **Figure S1**: mRNAsi correlates with clinical features. (A) Relationship between mRNAsi distribution and clinical features, including TCGA-Subtype, TP53 mutation, Mutation-Count, Stage, Tobacco-Smoking-History, the columns indicate the samples with mRNAsi ranked from low to high, and the rows indicate different characteristics. (B) Overview of the association between mRNAsi and patients with TMB and gene mutations. (C) Differences in clinical characteristics between the High mRNAsi and low mRNAsi groups. Columns indicate samples and rows indicate known clinical features. (D) Heat map showing the expression levels of DEGs between the two groups, red indicates high expression and blue indicates low expression.

**Additional file 3**. **Figure S2**: DEGs pathway analysis and CNV analysis. (A) Functional enrichment analysis of DEGs, including BP, CC and MF. (B) Scatter plot of KEGG pathway enrichment statistics. The circle size indicates the number of enriched genes (count), and the color shades indicate the size of log(q-value), the redder the color indicates the more significant. (C) Waterfall plot showing the 10 genes with the highest mutation frequency in DEGs, and different colors indicate different mutation types. (D) Circos plot showing the CNV of some DEGs, red color indicates amplification and blue color indicates deletion.

**Additional file 4**. **Figure S3**: The process of stemness subtype construction. (A) Consistency matrix plot showing clustering at K=2, which is the optimal number of clusters. (B) CDF plot showing the consensus distributions for each K, K=2-9. (C) Delta area showing the relative change in stability, K=2-9. (D) Heat map showing the expression of 217 DEGs, red indicates high expression and green indicates low expression. The top of the heat map shows the mRNAsi, TCGA subtype and immune subtype for each patient.

**Additional file 5**: **Figure S4**: Identification of a validation set of stemness subtypes based on DEGs. (A) Consistency matrix plot showing the clustering at k=2, which is the optimal number of clusters. (B) CDF plot showing the consensus distribution for each K, k=2-9. (C) Delta area showing the relative change in stability, k=2-9. (D) Survival analysis shows differences in DFS between Stemness Subtype I and Stemness Subtype II.

**Additional file 6**. **Figure S5**. Stemness subtype chemotherapy sensitivity analysis and potential compound identification. (A) Box plot showing the sensitivity of chemotherapeutic agents between stemness subtypes, red indicates Stemness Subtype I, green indicates Stemness Subtype II. (B) Scatter plot showing the relationship between compounds and MoA in Stemness Subtype I and Stemness Subtype II, rows indicate MoA, columns indicate compounds.

**Additional file 7**. **Figure S6**: Validation of the analysis of differences in clinical characteristics between subtypes, tumor immune infiltration, and tumor microenvironment. (A) Differences in clinical characteristics between Stemness Subtype I and Stemness Subtype II groups. (B) Comparison of the abundance of 22 immune cell types in immune subtypes. (C) Comparison of differences in the immune score, stromal score and tumor purity among different stemness subtypes by violin plot. * indicates P<0.05, ** indicates P<0.01, *** indicates P<0.001, and ns indicates no statistical significance. (D) Analysis of immune checkpoint gene differences, red indicates Stemness Subtype I, green indicates Stemness Subtype II. (E) Survival differences between two groups with high and low TIDE, green indicates low TIDE score, and red indicates High TIDE score. (F) Distribution of different histological samples between high and low TIDE groups, sky blue indicates ADC, brown indicates SQC.(G) Differences in TIDE between cancer types, green indicates ADC, yellow indicates SQC.(H) Box plot showing the sensitivity of chemotherapeutic drugs between stemness subtypes, red indicates Stemness Subtype I, green indicates Stemness Subtype II.

## Data Availability

The datasets supporting the findings of this study are available in the from The Cancer Genome Atlas (TCGA) (https://gdc.xenahubs.net) and GEO (https://www.ncbi.nlm.nih.gov/geo/) databases. Further inquiries can be directed to the corresponding author.

## Abbreviations

AUC: Area under the ROC curve
BP: Biological processes
CC: Cellular component
CDF: Cumulative distribution function
CMap: Connectivity map
CNV: Copy number variation
CSC: Cancer stem cell
DEG: Differentially expressed gene
ESTIMATE: Estimation of STromal and Immune cells in Malignant Tumor tissues using Expression data
FC: Fold change
GO: Gene ontology
GSVA: Gene set variation analysis
KEGG: Kyoto encyclopedia of genes and genomes
K–M: Kaplan–Meier
LASSO: Least absolute shrinkage and selection operator
MoA: Mode of action
MF: Molecular function
mRNAsi: Stemness index
NSCLC: Non-small cell lung cancer
OCLR: One-class logistic regression
OS: Overall survival
PCBC: Progenitor Cell Biology Consortium
RFB: Random forest and Boruta
ROC: Receiver operating characteristic
ssGSEA: Single sample gene set enrichment analysis
SVM-RFE: Support vector machine recursive feature elimination
TCGA: The Cancer Genome Atlas
TIME: Tumor immune microenvironment
TMB: Tumor mutation burden
XGBoost: Extreme gradient boosting
