# Supplementary material for: Machine learning-identified stemness features and constructed stemness-related subtype with prognosis, chemotherapy, and immunotherapy responses for non-small cell lung cancer patients
Source: Stem Cell Res Ther. 2023 Sep 7;14:238. doi: 10.1186/s13287-023-03406-4 (PMC10483786; doi:10.1186/s13287-023-03406-4)
Supplement: Supplementary file 1 — Additional file 1. Table S1: Patient clinical information of GSE30219. Table S2: Clinical information of 5 NSCLC patients. Table S3: Primers of Quantitative Real-time PCR. Table S4: The siRNA specific for ARTN mRNA. Table S5: 217 DEGs between high and low mRNAsi group. [file 13287_2023_3406_MOESM1_ESM.docx]

**Supplementary Table 1.** Patient clinical information of GSE30219.

|  | **ADC (N=85)** | **SQC (N=61)** | **Total (N=146)** |
| --- | --- | --- | --- |
| **Age** |  |  |  |
| Mean (SD) | 61.494 (9.281) | 63.508 (8.725) | 62.336 (9.077) |
| Range | 44.000 - 84.000 | 46.000 - 82.000 | 44.000 - 84.000 |
| **Gender** |  |  |  |
| F | 19 (22.4%) | 5 (8.2%) | 24 (16.4%) |
| M | 66 (77.6%) | 56 (91.8%) | 122 (83.6%) |
| **Stage** |  |  |  |
| Stage I | 81 (95.3%) | 50 (82.0%) | 131 (89.7%) |
| Stage II | 3 (3.5%) | 7 (11.5%) | 10 (6.8%) |
| Stage III | 1 (1.2%) | 4 (6.6%) | 5 (3.4%) |
| **Status** |  |  |  |
| ALIVE | 40 (47.1%) | 18 (29.5%) | 58 (39.7%) |
| DEAD | 45 (52.9%) | 43 (70.5%) | 88 (60.3%) |
| **Relapse** |  |  |  |
| N/A | 0 (0.0%) | 1 (1.6%) | 1 (0.7%) |
| Event | 27 (31.8%) | 22 (36.1%) | 49 (33.6%) |
| No Event | 58 (68.2%) | 38 (62.3%) | 96 (65.8%) |

**Supplementary Table 2.** Clinical information of 5 NSCLC patients.

| **Sample** | **Age**  **(Years)** | **Gender** | **Smoking history** | **TNM stage** | **Lymph node status** | **Histological type** | **Differentiated degree** |
| --- | --- | --- | --- | --- | --- | --- | --- |
| 1 | 78 | Female | No | III | Yes | Adenocarcinoma | Low |
| 2 | 59 | Male | Yes | II | No | Adenocarcinoma | Middle |
| 3 | 54 | Male | Yes | II | Yes | Adenocarcinoma | Low |
| 4 | 65 | Male | Yes | I | No | Adenocarcinoma | Low |
| 5 | 64 | Female | Yes | II | No | Squamous carcinoma | Low |

**Supplementary Table 3.** Primers of Quantitative Real-time PCR.

| **Gene Symbol** | **Forward Primer (5'to 3')** | **Reverse Primer (5'to 3')** |
| --- | --- | --- |
| ARTN | ATGAACACTACAGTGGCTGAGG | AGCTCCCATGAGTGAGTACAGG |
| SOX2 | TGGACAGTTACGCGCACAT | CGAGTAGGACATGCTGTAGGT |
| NANOG | CCCCAGCCTTTACTCTTCCTA | CCAGGTTGAATTGTTCCAGGTC |
| CD44 | CTGCCGCTTTGCAGGTGTA | CATTGTGGGCAAGGTGCTATT |
| POU5F1 | CTTGAATCCCGAATGGAAAGGG | GTGTATATCCCAGGGTGATCCTC |

**Supplementary Table 4.** The siRNA specific for ARTN mRNA.

| Gene Symbol | Sequence of SiRNAs | |
| --- | --- | --- |
|  | Sense（5'-3'） | Anti-sense（5'-3'） |
| ARTN-siRNA-NC | UUCUCCGAACGUGUCACGUTT | ACGUGACACGUUCGGAGAATT |
| ARTN-siRNA-1 | UCAUGGACGUCAACAGCACTT | GUGCUGUUGACGUCCAUGATT |
| ARTN-siRNA-2 | GGUGGGUGAUGGAUAUCAUTT | AUGAUAUCCAUCACCCACCTT |

**Supplementary Table 5.** 217 DEGs between high and low mRNAsi group.

| **Genes** | **logFC** | **AveExpr** | **t** | **P.Value** | **adj.P.Val** | **B** |
| --- | --- | --- | --- | --- | --- | --- |
| *NAPSA* | -3.841307405 | 11.43235103 | -16.53589354 | 1.42E-54 | 3.51E-53 | 113.2507017 |
| *SFTA2* | -3.643345994 | 7.741269783 | -16.11867391 | 3.20E-52 | 6.83E-51 | 107.8656544 |
| *MUC21* | -3.634751557 | 6.293130266 | -15.84166645 | 1.11E-50 | 2.24E-49 | 104.3353258 |
| *SFTA3* | -3.422002727 | 8.119871358 | -15.35760056 | 5.05E-48 | 8.83E-47 | 98.25554542 |
| *SLC34A2* | -3.402365837 | 12.41371348 | -17.58286168 | 1.27E-60 | 4.35E-59 | 127.1032688 |
| *C16orf89* | -3.353120161 | 8.503346309 | -16.08995635 | 4.63E-52 | 9.79E-51 | 107.4979709 |
| *SFTPB* | -3.281088835 | 14.05326895 | -13.48030862 | 3.19E-38 | 3.27E-37 | 75.84040225 |
| *HNF1B* | -3.185248839 | 6.123012894 | -16.21397919 | 9.35E-53 | 2.06E-51 | 109.0886623 |
| *CAPN8* | -3.177717144 | 6.608065059 | -16.63103254 | 4.09E-55 | 1.03E-53 | 114.4897852 |
| *CTSE* | -3.149404219 | 9.127235236 | -14.65837997 | 2.82E-44 | 3.97E-43 | 89.68324777 |
| *C4BPA* | -3.031199234 | 8.773918455 | -13.76596256 | 1.17E-39 | 1.30E-38 | 79.12471722 |
| *FOLR1* | -3.013839623 | 8.429166339 | -15.41551189 | 2.45E-48 | 4.34E-47 | 98.97678331 |
| *PGC* | -3.009171258 | 7.221248081 | -10.96230605 | 1.66E-26 | 9.28E-26 | 49.08880696 |
| *NKX2-1* | -3.000187551 | 8.581256841 | -14.81175284 | 4.34E-45 | 6.44E-44 | 91.54169399 |
| *SFTPA1* | -2.978835182 | 11.51271555 | -11.50975163 | 6.68E-29 | 4.23E-28 | 54.55040643 |
| *XAGE1D* | -2.959493826 | 6.281418701 | -9.948415868 | 2.58E-22 | 1.18E-21 | 39.53584813 |
| *SFTPA2* | -2.913562186 | 12.67458509 | -11.4261259 | 1.57E-28 | 9.76E-28 | 53.70276465 |
| *SPINK1* | -2.905788542 | 5.676400295 | -11.77778741 | 4.17E-30 | 2.83E-29 | 57.29911588 |
| *MUC5B* | -2.895381908 | 9.14174936 | -11.32819452 | 4.25E-28 | 2.59E-27 | 52.71618902 |
| *HABP2* | -2.883513159 | 5.485944587 | -15.54480755 | 4.81E-49 | 8.80E-48 | 100.593108 |
| *CYP2B7P1* | -2.863756105 | 7.947353297 | -15.06817857 | 1.85E-46 | 2.95E-45 | 94.6765334 |
| *MSLN* | -2.849497349 | 8.814501624 | -12.30279677 | 1.59E-32 | 1.21E-31 | 62.82108057 |
| *PCDP1* | -2.829689739 | 5.369479035 | -17.84658297 | 3.54E-62 | 1.33E-60 | 130.6648508 |
| *ROS1* | -2.828803233 | 8.086447835 | -17.02347726 | 2.30E-57 | 6.50E-56 | 119.6433779 |
| *GGTLC1* | -2.810948185 | 4.809087598 | -16.8233529 | 3.25E-56 | 8.76E-55 | 117.0068877 |
| *PIGR* | -2.802843047 | 10.52856727 | -13.73293603 | 1.71E-39 | 1.90E-38 | 78.74257059 |
| *CEACAM6* | -2.756614026 | 12.35111545 | -14.92161194 | 1.13E-45 | 1.72E-44 | 92.88052642 |
| *LMO3* | -2.73823941 | 8.345671604 | -14.71985519 | 1.33E-44 | 1.92E-43 | 90.42665079 |
| *SCGB3A1* | -2.718232735 | 7.87698937 | -12.22331311 | 3.74E-32 | 2.78E-31 | 61.97353896 |
| *SLC1A7* | -2.710434671 | 4.757508563 | -15.9433932 | 3.04E-51 | 6.26E-50 | 105.6275348 |
| *SFTPC* | -2.71010408 | 9.163210384 | -8.989319068 | 1.19E-18 | 4.48E-18 | 31.20580738 |
| *SPDEF* | -2.648158896 | 6.808822392 | -14.72725373 | 1.22E-44 | 1.76E-43 | 90.5162552 |
| *AMY1A* | -2.636893609 | 6.956143504 | -13.75396609 | 1.34E-39 | 1.49E-38 | 78.98583453 |
| *C4orf31* | -2.621066946 | 8.212479331 | -18.21187429 | 2.37E-64 | 1.04E-62 | 135.6434709 |
| *CHIA* | -2.615422924 | 3.825996457 | -14.04245626 | 4.54E-41 | 5.42E-40 | 82.34848678 |
| *VSTM2L* | -2.611281494 | 7.8876156 | -16.43051658 | 5.62E-54 | 1.34E-52 | 111.8830545 |
| *PLUNC* | -2.611011578 | 6.198525738 | -8.930831086 | 1.94E-18 | 7.25E-18 | 30.72090555 |
| *SCTR* | -2.610070923 | 5.393223031 | -14.35436724 | 1.11E-42 | 1.43E-41 | 86.03685212 |
| *B3GNT6* | -2.60700076 | 4.065654134 | -13.07612039 | 3.17E-36 | 2.94E-35 | 71.2756098 |
| *GPR39* | -2.582342558 | 7.117918848 | -18.89440672 | 1.80E-68 | 1.05E-66 | 145.0804548 |
| *ELFN2* | -2.568866918 | 5.687736811 | -16.81465344 | 3.65E-56 | 9.79E-55 | 116.8926753 |
| *SFTPD* | -2.560970248 | 9.17270561 | -13.41008391 | 7.13E-38 | 7.21E-37 | 75.04031108 |
| *COMP* | -2.559832902 | 7.715867913 | -17.07630886 | 1.14E-57 | 3.27E-56 | 120.3422873 |
| *RORC* | -2.555427343 | 7.695488681 | -15.60808593 | 2.16E-49 | 4.01E-48 | 101.3871759 |
| *DMBT1* | -2.555381439 | 8.712808022 | -11.66458033 | 1.35E-29 | 8.90E-29 | 56.13228272 |
| *DPCR1* | -2.531879108 | 4.169140797 | -14.66542407 | 2.59E-44 | 3.66E-43 | 89.76832769 |
| *CYP4B1* | -2.525994078 | 7.872736614 | -13.37389523 | 1.08E-37 | 1.08E-36 | 74.62914206 |
| *STK32A* | -2.513138912 | 4.244500098 | -16.34058717 | 1.81E-53 | 4.19E-52 | 110.7199012 |
| *LOC84740* | -2.509984597 | 7.287058957 | -11.2644735 | 8.10E-28 | 4.87E-27 | 52.07778946 |
| *SLC44A4* | -2.507163493 | 9.018564764 | -15.57635495 | 3.23E-49 | 5.94E-48 | 100.9887428 |
| *LRRN4* | -2.467140587 | 5.538555955 | -15.00132006 | 4.23E-46 | 6.59E-45 | 93.85587286 |
| *C20orf56* | -2.466300501 | 5.101780955 | -13.396188 | 8.37E-38 | 8.41E-37 | 74.88233648 |
| *SFTA1P* | -2.463441014 | 5.491218209 | -15.82750654 | 1.34E-50 | 2.67E-49 | 104.1558508 |
| *DPP4* | -2.457267392 | 8.984909301 | -17.48594893 | 4.70E-60 | 1.56E-58 | 125.8015234 |
| *PEBP4* | -2.454544571 | 5.155939665 | -12.88156584 | 2.80E-35 | 2.46E-34 | 69.11356343 |
| *SLC26A9* | -2.452659139 | 7.028052756 | -15.01640058 | 3.51E-46 | 5.50E-45 | 94.04077754 |
| *FOXA2* | -2.435995642 | 6.443062205 | -13.92877002 | 1.73E-40 | 2.01E-39 | 81.017697 |
| *MMP13* | -2.435089459 | 7.0537375 | -12.17601171 | 6.20E-32 | 4.57E-31 | 61.47109683 |
| *ARSE* | -2.424138148 | 6.204631841 | -14.43530211 | 4.19E-43 | 5.53E-42 | 87.00269784 |
| *SLC22A3* | -2.417543994 | 7.599688041 | -18.48732818 | 5.27E-66 | 2.55E-64 | 139.4313947 |
| *AGR3* | -2.413005517 | 7.378456841 | -12.57657493 | 8.12E-34 | 6.58E-33 | 65.77134788 |
| *SCN7A* | -2.406567965 | 5.551118209 | -18.67210826 | 4.03E-67 | 2.08E-65 | 141.9881765 |
| *ADH1B* | -2.399032503 | 7.407689665 | -12.87089956 | 3.15E-35 | 2.76E-34 | 68.99570279 |
| *SORCS2* | -2.398738529 | 7.090493159 | -21.12957489 | 1.97E-82 | 4.54E-80 | 177.0707947 |
| *MLPH* | -2.392905291 | 9.715217175 | -16.7765563 | 6.03E-56 | 1.60E-54 | 116.3929028 |
| *CLDN2* | -2.390907987 | 5.543415305 | -13.11605169 | 2.02E-36 | 1.89E-35 | 71.72221387 |
| *KRT7* | -2.389429734 | 11.67036329 | -14.4322262 | 4.35E-43 | 5.73E-42 | 86.96592557 |
| *SLC4A4* | -2.382349343 | 7.295041831 | -17.45289833 | 7.33E-60 | 2.40E-58 | 125.358464 |
| *BCL2L15* | -2.374922152 | 5.668783858 | -15.44192789 | 1.76E-48 | 3.14E-47 | 99.30633156 |
| *KLHDC7A* | -2.371328929 | 5.552876722 | -15.1853421 | 4.33E-47 | 7.17E-46 | 96.12023121 |
| *PNMA2* | -2.355476481 | 7.320218159 | -20.18205939 | 1.97E-76 | 2.36E-74 | 163.3220194 |
| *SUSD2* | -2.34128755 | 9.270790748 | -17.32263131 | 4.23E-59 | 1.32E-57 | 123.6165735 |
| *C7* | -2.335695935 | 8.92485374 | -15.2870784 | 1.22E-47 | 2.07E-46 | 97.37953291 |
| *BEAN* | -2.330906908 | 5.503493947 | -19.84327574 | 2.58E-74 | 2.39E-72 | 158.4705718 |
| *AQP4* | -2.325115099 | 8.029532776 | -11.25373029 | 9.03E-28 | 5.41E-27 | 51.97043249 |
| *VSIG2* | -2.313592695 | 6.188165059 | -14.06045865 | 3.67E-41 | 4.41E-40 | 82.55988892 |
| *COL4A3* | -2.302912281 | 6.258502264 | -15.91534666 | 4.35E-51 | 8.91E-50 | 105.2707711 |
| *AQP5* | -2.302669049 | 6.144327707 | -10.71198021 | 1.92E-25 | 1.02E-24 | 46.66121652 |
| *STEAP4* | -2.295601287 | 9.793511909 | -16.73172412 | 1.09E-55 | 2.84E-54 | 115.8055981 |
| *APOH* | -2.285578628 | 3.12721373 | -12.43367722 | 3.86E-33 | 3.02E-32 | 64.2255103 |
| *GJB1* | -2.276702064 | 4.93234247 | -11.12402709 | 3.32E-27 | 1.92E-26 | 50.68061106 |
| *MMP28* | -2.274939011 | 7.180565896 | -17.3204153 | 4.35E-59 | 1.36E-57 | 123.5870027 |
| *OGN* | -2.257620713 | 4.944119882 | -18.24353636 | 1.53E-64 | 6.83E-63 | 136.0774207 |
| *SCGB3A2* | -2.253274789 | 8.702733809 | -9.325688253 | 6.69E-20 | 2.68E-19 | 34.04660756 |
| *UBXN10* | -2.245269009 | 6.546815896 | -17.15588415 | 3.94E-58 | 1.17E-56 | 121.3972446 |
| *SHISA3* | -2.237029228 | 4.471628002 | -13.41118544 | 7.05E-38 | 7.13E-37 | 75.05283865 |
| *FMO5* | -2.236289235 | 7.370756447 | -14.88548334 | 1.76E-45 | 2.66E-44 | 92.43953382 |
| *TMC5* | -2.234643052 | 9.261598622 | -14.10824595 | 2.08E-41 | 2.53E-40 | 83.12193903 |
| *ITGA8* | -2.2226023 | 5.127077608 | -19.2740435 | 8.57E-71 | 6.07E-69 | 150.4014942 |
| *XIST* | -2.220141075 | 6.023521703 | -6.424491449 | 2.03E-10 | 4.90E-10 | 12.58608072 |
| *KNDC1* | -2.207678912 | 5.610827904 | -15.16716895 | 5.43E-47 | 8.95E-46 | 95.89583829 |
| *KIAA0408* | -2.196671242 | 3.391218947 | -12.32146083 | 1.30E-32 | 9.91E-32 | 63.02068682 |
| *FCER1A* | -2.192472444 | 4.821323278 | -17.1380373 | 4.99E-58 | 1.47E-56 | 121.1604077 |
| *MUC1* | -2.186757437 | 12.36217667 | -16.77927787 | 5.82E-56 | 1.55E-54 | 116.4285841 |
| *C20orf114* | -2.182143226 | 7.845504577 | -8.549473012 | 4.49E-17 | 1.55E-16 | 27.6258852 |
| *C1QTNF7* | -2.168420773 | 4.648557923 | -22.92273799 | 4.63E-94 | 3.61E-91 | 203.7260101 |
| *TMPRSS6* | -2.162471769 | 4.604886467 | -14.29165826 | 2.35E-42 | 2.99E-41 | 85.29097943 |
| *ATOH8* | -2.161204748 | 7.13643873 | -16.10548917 | 3.79E-52 | 8.06E-51 | 107.6967962 |
| *GPR116* | -2.142618034 | 10.37888725 | -20.315535 | 2.86E-77 | 3.64E-75 | 165.2430528 |
| *TMPRSS2* | -2.141032218 | 9.282606348 | -15.79273546 | 2.08E-50 | 4.13E-49 | 103.7155429 |
| *CRYM* | -2.133591403 | 5.711324803 | -11.56349447 | 3.84E-29 | 2.47E-28 | 55.09765863 |
| *ACSL5* | -2.126964096 | 9.975246752 | -17.4333811 | 9.54E-60 | 3.09E-58 | 125.0970376 |
| *ABCA8* | -2.1207 | 5.339129478 | -16.460081 | 3.83E-54 | 9.17E-53 | 112.2662505 |
| *CLDN18* | -2.112738581 | 6.475179528 | -9.821363139 | 8.22E-22 | 3.63E-21 | 38.39201581 |
| *EPHA10* | -2.110264204 | 5.538766093 | -13.91483778 | 2.04E-40 | 2.36E-39 | 80.85511291 |
| *LOC100130933* | -2.107220662 | 3.724506053 | -15.18601188 | 4.30E-47 | 7.12E-46 | 96.12850459 |
| *CLDN9* | -2.104609003 | 4.842922785 | -14.53547541 | 1.25E-43 | 1.71E-42 | 88.20307314 |
| *GPR133* | -2.102453748 | 7.237541339 | -16.17731515 | 1.50E-52 | 3.27E-51 | 108.617667 |
| *KIF12* | -2.097821102 | 4.97356122 | -13.5101983 | 2.26E-38 | 2.33E-37 | 76.18182647 |
| *LRRC31* | -2.095571928 | 3.013986171 | -11.62400445 | 2.06E-29 | 1.34E-28 | 55.71615706 |
| *FGG* | -2.092846032 | 7.583783071 | -7.532580753 | 1.10E-13 | 3.16E-13 | 19.95197033 |
| *RASGRF1* | -2.091534674 | 5.971438976 | -15.08517718 | 1.50E-46 | 2.42E-45 | 94.88555321 |
| *HOPX* | -2.091090026 | 10.52850827 | -16.5237469 | 1.67E-54 | 4.09E-53 | 113.0927982 |
| *CST6* | -2.090566168 | 6.104733858 | -15.04907379 | 2.35E-46 | 3.73E-45 | 94.44179378 |
| *KCNQ3* | -2.075074325 | 4.778080906 | -15.52795849 | 5.95E-49 | 1.09E-47 | 100.3820064 |
| *ELN* | -2.074122738 | 9.682943553 | -19.07260905 | 1.47E-69 | 9.41E-68 | 147.5719572 |
| *C1orf116* | -2.062885497 | 10.4512906 | -18.20523385 | 2.60E-64 | 1.14E-62 | 135.5525077 |
| *C6* | -2.056449623 | 3.402840797 | -13.85730788 | 4.01E-40 | 4.57E-39 | 80.18493167 |
| *HKDC1* | -2.047409709 | 6.497932431 | -13.0241443 | 5.68E-36 | 5.20E-35 | 70.69574465 |
| *PLA2G1B* | -2.046181926 | 3.04359252 | -13.33907893 | 1.61E-37 | 1.60E-36 | 74.23429976 |
| *CACNA2D2* | -2.044393733 | 7.555785482 | -13.18915249 | 8.84E-37 | 8.45E-36 | 72.5422963 |
| *SCEL* | -2.042611859 | 8.131054675 | -13.22083903 | 6.17E-37 | 5.96E-36 | 72.89877332 |
| *CRTAC1* | -2.041776021 | 7.057903593 | -12.63631486 | 4.22E-34 | 3.45E-33 | 66.42142522 |
| *C13orf30* | -2.041715784 | 3.911214222 | -12.43585402 | 3.77E-33 | 2.96E-32 | 64.2489612 |
| *ADAMTS16* | -2.040325514 | 5.541023278 | -17.11452999 | 6.83E-58 | 1.99E-56 | 120.8486608 |
| *ISM1* | -2.03506079 | 6.570412697 | -22.3043802 | 5.16E-90 | 2.68E-87 | 194.4491151 |
| *CRLF1* | -2.027234921 | 7.340755413 | -11.2039249 | 1.49E-27 | 8.82E-27 | 51.47376856 |
| *HPN* | -2.024209854 | 7.856697293 | -11.59588208 | 2.75E-29 | 1.78E-28 | 55.4283989 |
| *HHLA2* | -2.017914718 | 4.506337106 | -10.53957384 | 1.01E-24 | 5.20E-24 | 45.01526117 |
| *MAMDC2* | -2.015690694 | 5.993684449 | -16.6374897 | 3.76E-55 | 9.54E-54 | 114.57403 |
| *PAEP* | -2.014746935 | 4.178302854 | -8.403887993 | 1.44E-16 | 4.86E-16 | 26.47514539 |
| *MFAP4* | -2.012340576 | 9.808708612 | -21.74026801 | 2.36E-86 | 8.37E-84 | 186.0618375 |
| *GLB1L3* | -2.012309943 | 3.360338386 | -10.15929739 | 3.69E-23 | 1.75E-22 | 41.46097828 |
| *ALOX15B* | -2.010557269 | 8.158399163 | -14.96836572 | 6.35E-46 | 9.80E-45 | 93.45222615 |
| *ABCC6* | -2.009550602 | 6.743764911 | -16.38009739 | 1.08E-53 | 2.54E-52 | 111.2304726 |
| *HLA-DQB2* | -2.008164644 | 7.866076476 | -15.75272125 | 3.46E-50 | 6.73E-49 | 103.2095658 |
| *TOX3* | -2.004484647 | 6.020532726 | -9.752771653 | 1.53E-21 | 6.67E-21 | 37.77954785 |
| *TMEM130* | -2.004379646 | 6.250162598 | -16.62994617 | 4.15E-55 | 1.05E-53 | 114.4756134 |
| *ACOX2* | -2.00032138 | 6.32795502 | -18.72889155 | 1.83E-67 | 9.58E-66 | 142.7763756 |
| *STXBP5L* | 2.009218819 | 2.305805069 | 12.70142531 | 2.06E-34 | 1.72E-33 | 67.13249425 |
| *KRT31* | 2.0113042 | 2.625889419 | 9.72496387 | 1.96E-21 | 8.52E-21 | 37.53225901 |
| *DLX6AS* | 2.015160686 | 2.896642963 | 14.14443711 | 1.36E-41 | 1.66E-40 | 83.54845299 |
| *KRT14* | 2.046625539 | 7.290703346 | 6.093847571 | 1.56E-09 | 3.58E-09 | 10.59640563 |
| *FAM83F* | 2.058110396 | 6.645289715 | 12.0936437 | 1.49E-31 | 1.08E-30 | 60.59963916 |
| *GDA* | 2.059784163 | 4.932866142 | 9.967688723 | 2.16E-22 | 9.88E-22 | 39.71041471 |
| *ZIC5* | 2.064296835 | 3.598440207 | 11.32703617 | 4.30E-28 | 2.62E-27 | 52.70455903 |
| *PART1* | 2.065698585 | 3.375862451 | 11.67007798 | 1.28E-29 | 8.44E-29 | 56.18874895 |
| *CALB1* | 2.070814538 | 3.048756152 | 9.50228679 | 1.42E-20 | 5.92E-20 | 35.57323723 |
| *ARTN* | 2.072085124 | 6.04855625 | 15.02577307 | 3.13E-46 | 4.92E-45 | 94.15575463 |
| *CNTNAP2* | 2.075058318 | 6.422257579 | 10.00263111 | 1.57E-22 | 7.22E-22 | 40.02761764 |
| *ALDH3A1* | 2.076772655 | 8.616632283 | 8.908440075 | 2.34E-18 | 8.70E-18 | 30.53598554 |
| *MRAP2* | 2.090291396 | 5.14733686 | 11.8277413 | 2.47E-30 | 1.69E-29 | 57.81671497 |
| *DMRT2* | 2.111360292 | 4.741442667 | 11.24902919 | 9.46E-28 | 5.66E-27 | 51.92347936 |
| *SPRR3* | 2.132525053 | 5.218515453 | 7.041405624 | 3.50E-12 | 9.28E-12 | 16.5559521 |
| *BNC1* | 2.143996171 | 5.264261959 | 8.267587566 | 4.24E-16 | 1.39E-15 | 25.41338723 |
| *DLX5* | 2.162214009 | 5.487093307 | 12.5480012 | 1.11E-33 | 8.94E-33 | 65.46120929 |
| *IGF2BP1* | 2.170002243 | 4.657140305 | 9.981902798 | 1.90E-22 | 8.70E-22 | 39.83933848 |
| *GABRA3* | 2.200978776 | 4.024995571 | 11.6718443 | 1.26E-29 | 8.28E-29 | 56.20689519 |
| *SPRR2E* | 2.203359608 | 3.480164911 | 8.36701406 | 1.94E-16 | 6.47E-16 | 26.18641101 |
| *PKP1* | 2.219211819 | 10.03069759 | 8.20251848 | 7.06E-16 | 2.29E-15 | 24.91185768 |
| *TP63* | 2.221644932 | 9.429105266 | 9.130208439 | 3.60E-19 | 1.39E-18 | 32.38492264 |
| *SPRR1A* | 2.249269653 | 4.941791781 | 8.013770929 | 3.04E-15 | 9.52E-15 | 23.47673381 |
| *TMPRSS11A* | 2.260630493 | 3.147726033 | 9.905286347 | 3.83E-22 | 1.73E-21 | 39.14620323 |
| *GBP6* | 2.268237872 | 6.983994439 | 9.808396239 | 9.24E-22 | 4.07E-21 | 38.27595953 |
| *MAGEA11* | 2.285559076 | 2.722438189 | 10.14656296 | 4.15E-23 | 1.96E-22 | 41.3437901 |
| *FAM83B* | 2.291175583 | 6.008567815 | 11.2574727 | 8.69E-28 | 5.22E-27 | 52.0078214 |
| *HAP1* | 2.300178593 | 5.158293898 | 13.11371982 | 2.07E-36 | 1.94E-35 | 71.69610696 |
| *MAGEA3* | 2.335064122 | 4.117126969 | 8.463871385 | 8.94E-17 | 3.04E-16 | 26.94719154 |
| *PNCK* | 2.338511164 | 5.26497815 | 11.61275379 | 2.31E-29 | 1.50E-28 | 55.60097224 |
| *NR0B1* | 2.342026963 | 2.656936122 | 10.48621252 | 1.69E-24 | 8.56E-24 | 44.51016219 |
| *C12orf56* | 2.360727372 | 4.102512795 | 13.92920699 | 1.72E-40 | 2.01E-39 | 81.02279809 |
| *SOX2OT* | 2.394631977 | 4.434837844 | 15.08466994 | 1.51E-46 | 2.44E-45 | 94.8793139 |
| *NTRK2* | 2.401109138 | 8.203896998 | 10.06030344 | 9.23E-23 | 4.29E-22 | 40.55315025 |
| *WDR72* | 2.426604355 | 7.624532431 | 11.99192206 | 4.40E-31 | 3.11E-30 | 59.52953018 |
| *A2ML1* | 2.427900021 | 4.999020768 | 8.794686344 | 6.04E-18 | 2.19E-17 | 29.60266966 |
| *FAM83C* | 2.434029147 | 3.5354125 | 10.31223105 | 8.80E-24 | 4.31E-23 | 42.87767762 |
| *AKR1C1* | 2.450019289 | 10.92768105 | 11.62589266 | 2.02E-29 | 1.32E-28 | 55.73549697 |
| *ADAM23* | 2.462312639 | 5.952562156 | 10.96391087 | 1.63E-26 | 9.14E-26 | 49.10451293 |
| *HOXD11* | 2.463782452 | 3.391882726 | 13.05637406 | 3.95E-36 | 3.64E-35 | 71.05511856 |
| *MAGEA6* | 2.474512682 | 4.48312003 | 8.608474879 | 2.78E-17 | 9.74E-17 | 28.09712244 |
| *LOC642587* | 2.483013544 | 6.347494783 | 10.22839165 | 1.93E-23 | 9.31E-23 | 42.09890196 |
| *UPK1B* | 2.493843203 | 4.922403445 | 8.484051884 | 7.60E-17 | 2.59E-16 | 27.10665947 |
| *FOXE1* | 2.507384128 | 6.264858022 | 10.17256162 | 3.26E-23 | 1.55E-22 | 41.58316935 |
| *SOX2* | 2.519563574 | 8.9540281 | 12.83634735 | 4.63E-35 | 4.00E-34 | 68.6143887 |
| *CLCA2* | 2.532405719 | 7.047832874 | 8.199523109 | 7.23E-16 | 2.35E-15 | 24.8888539 |
| *SLC6A15* | 2.550358605 | 4.173726919 | 11.3759125 | 2.62E-28 | 1.61E-27 | 53.19608679 |
| *CYP4F11* | 2.588063988 | 6.621598917 | 10.74808405 | 1.35E-25 | 7.24E-25 | 47.00859491 |
| *NKAIN2* | 2.616983043 | 4.011996358 | 14.44411607 | 3.77E-43 | 4.99E-42 | 87.10809644 |
| *RHCG* | 2.637203859 | 5.249584006 | 9.940856158 | 2.77E-22 | 1.26E-21 | 39.46745082 |
| *LASS3* | 2.63801841 | 4.140805463 | 10.23933359 | 1.75E-23 | 8.42E-23 | 42.20024771 |
| *CACNA1B* | 2.650157204 | 4.113666732 | 12.92373528 | 1.75E-35 | 1.55E-34 | 69.58021507 |
| *SERPINB13* | 2.653273014 | 4.978848819 | 8.731388201 | 1.02E-17 | 3.65E-17 | 29.08778454 |
| *KRT6C* | 2.660224155 | 7.26208253 | 8.423159431 | 1.24E-16 | 4.18E-16 | 26.62648609 |
| *DLX6* | 2.698150687 | 4.194987008 | 14.80667171 | 4.62E-45 | 6.82E-44 | 91.47992508 |
| *SERPINB5* | 2.764677503 | 8.178052165 | 10.55894006 | 8.43E-25 | 4.33E-24 | 45.19908365 |
| *AKR1C2* | 2.767989689 | 10.17811924 | 11.62211683 | 2.10E-29 | 1.37E-28 | 55.69682555 |
| *SPRR2A* | 2.780089553 | 5.050514321 | 9.108812664 | 4.32E-19 | 1.66E-18 | 32.20485655 |
| *GPX2* | 2.79602099 | 9.176622785 | 11.36640837 | 2.89E-28 | 1.77E-27 | 53.10037958 |
| *UGT1A9* | 2.803248729 | 4.468928051 | 12.44680342 | 3.35E-33 | 2.64E-32 | 64.36696629 |
| *MAGEA9B* | 2.841914581 | 3.695683415 | 10.7083635 | 1.99E-25 | 1.06E-24 | 46.62646912 |
| *KRT6B* | 2.853855231 | 8.333427411 | 8.672161577 | 1.66E-17 | 5.87E-17 | 28.60891621 |
| *KRT13* | 2.874013283 | 7.092571801 | 8.937351791 | 1.84E-18 | 6.87E-18 | 30.77483245 |
| *DAPL1* | 2.902280996 | 4.906645866 | 11.64556699 | 1.65E-29 | 1.08E-28 | 55.9371536 |
| *UGT1A6* | 2.902499275 | 7.61605374 | 10.87248401 | 4.01E-26 | 2.21E-25 | 48.2126313 |
| *HOXD13* | 2.916022618 | 3.159820522 | 14.16233863 | 1.10E-41 | 1.35E-40 | 83.75969355 |
| *MAGEA4* | 2.920468706 | 4.323762992 | 9.559155215 | 8.62E-21 | 3.62E-20 | 36.06994564 |
| *KRT6A* | 2.927777144 | 10.64949936 | 8.258579316 | 4.56E-16 | 1.49E-15 | 25.34374822 |
| *PRAME* | 2.998377719 | 7.292164469 | 12.68701503 | 2.41E-34 | 2.00E-33 | 66.97489135 |
| *DSC3* | 3.097466185 | 7.766155413 | 10.50547392 | 1.41E-24 | 7.15E-24 | 44.6922461 |
| *UGT1A7* | 3.104337646 | 2.838925886 | 12.52205242 | 1.47E-33 | 1.18E-32 | 65.1800087 |
| *CYP4F3* | 3.121747807 | 5.178045817 | 13.70597884 | 2.34E-39 | 2.58E-38 | 78.43111845 |
| *KRT5* | 3.215423269 | 10.53128863 | 8.746357184 | 9.00E-18 | 3.24E-17 | 29.20925797 |
| *ADH7* | 3.288141348 | 5.299855758 | 11.39308364 | 2.20E-28 | 1.35E-27 | 53.36915801 |
| *AKR1B10* | 3.384056093 | 7.943056939 | 11.7245792 | 7.26E-30 | 4.86E-29 | 56.74962689 |
| *DSG3* | 3.589607857 | 6.733428642 | 10.17661014 | 3.14E-23 | 1.49E-22 | 41.6204904 |
| *NTS* | 3.647656482 | 5.849193799 | 12.3516119 | 9.39E-33 | 7.20E-32 | 63.34361548 |
| *CALML3* | 4.107726279 | 6.769539567 | 11.85575754 | 1.84E-30 | 1.27E-29 | 58.10773242 |
