# Supplementary figures and images for: Machine learning-identified stemness features and constructed stemness-related subtype with prognosis, chemotherapy, and immunotherapy responses for non-small cell lung cancer patients
Source: Stem Cell Res Ther. 2023 Sep 7;14:238. doi: 10.1186/s13287-023-03406-4 (PMC10483786; doi:10.1186/s13287-023-03406-4)

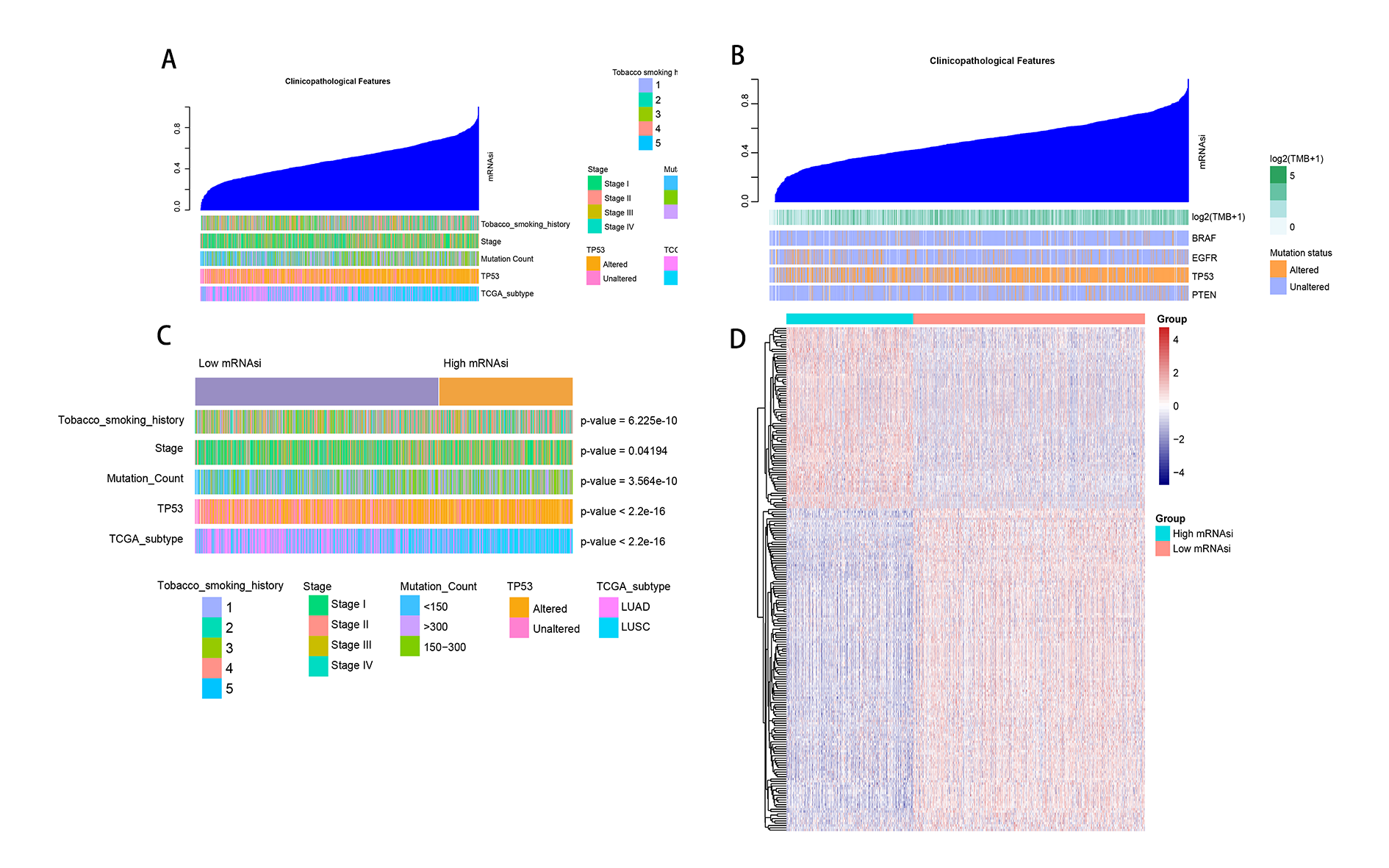

Supplement: Supplementary file 2 — Additional file 2. Figure S1: mRNAsi correlates with clinical features. (A) Relationship between mRNAsi distribution and clinical features, including TCGA-Subtype, TP53 mutation, Mutation-Count, Stage, Tobacco-Smoking-History, the columns indicate the samples with mRNAsi ranked from low to high, and the rows indicate different characteristics. (B) Overview of the association between mRNAsi and patients with TMB and gene mutations. (C) Differences in clinical characteristics between the High mRNAsi and low mRNAsi groups. Columns indicate samples and rows indicate known clinical features. (D) Heat map showing the expression levels of DEGs between the two groups, red indicates high expression and blue indicates low expression. [file 13287_2023_3406_MOESM2_ESM.tif]

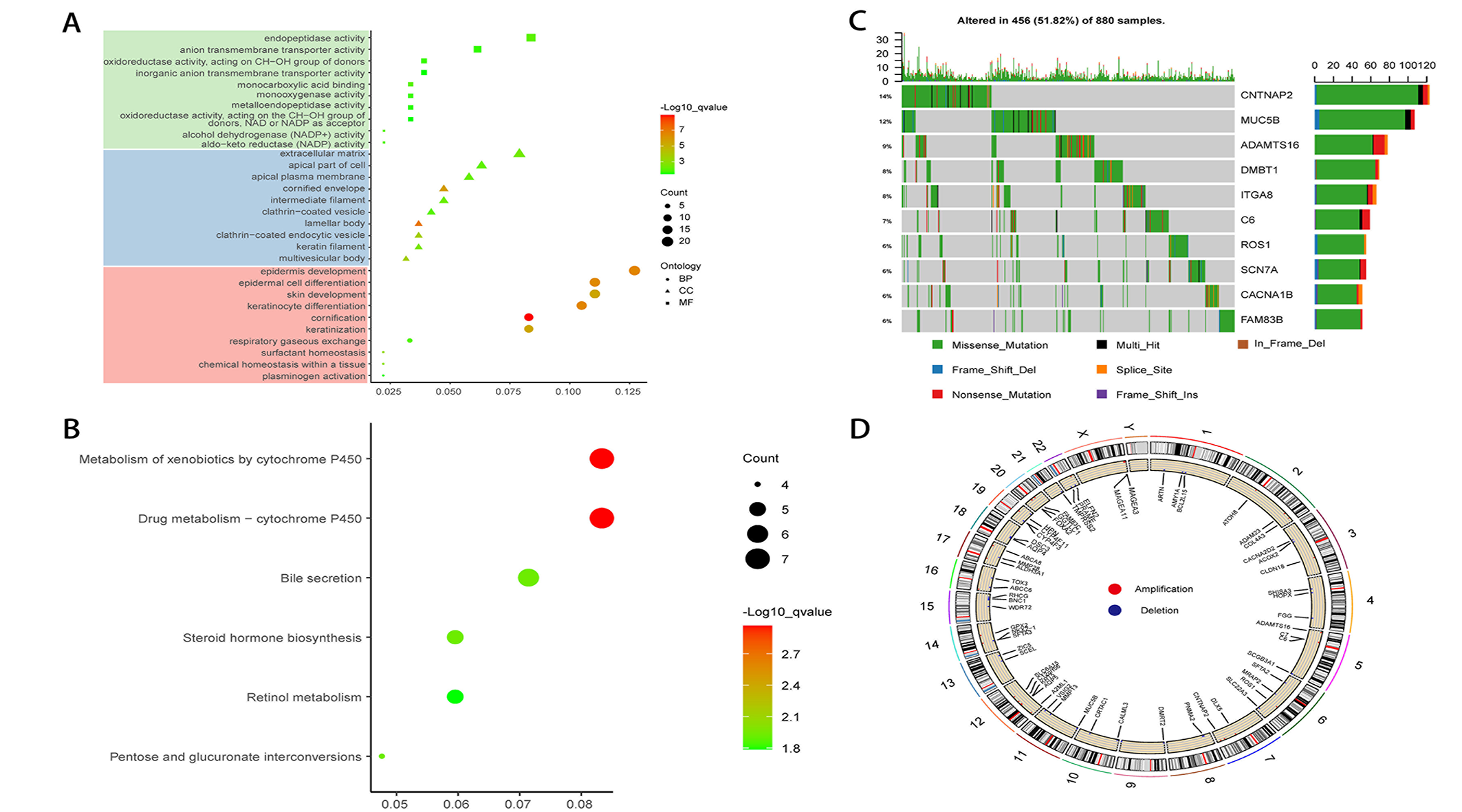

Supplement: Supplementary file 3 — Additional file 3. Figure S2: DEGs pathway analysis and CNV analysis. (A) Functional enrichment analysis of DEGs, including BP, CC and MF. (B) Scatter plot of KEGG pathway enrichment statistics. The circle size indicates the number of enriched genes (count), and the color shades indicate the size of log(q-value), the redder the color indicates the more significant. (C) Waterfall plot showing the 10 genes with the highest mutation frequency in DEGs, and different colors indicate different mutation types. (D) Circos plot showing the CNV of some DEGs, red color indicates amplification and blue color indicates deletion. [file 13287_2023_3406_MOESM3_ESM.tif]

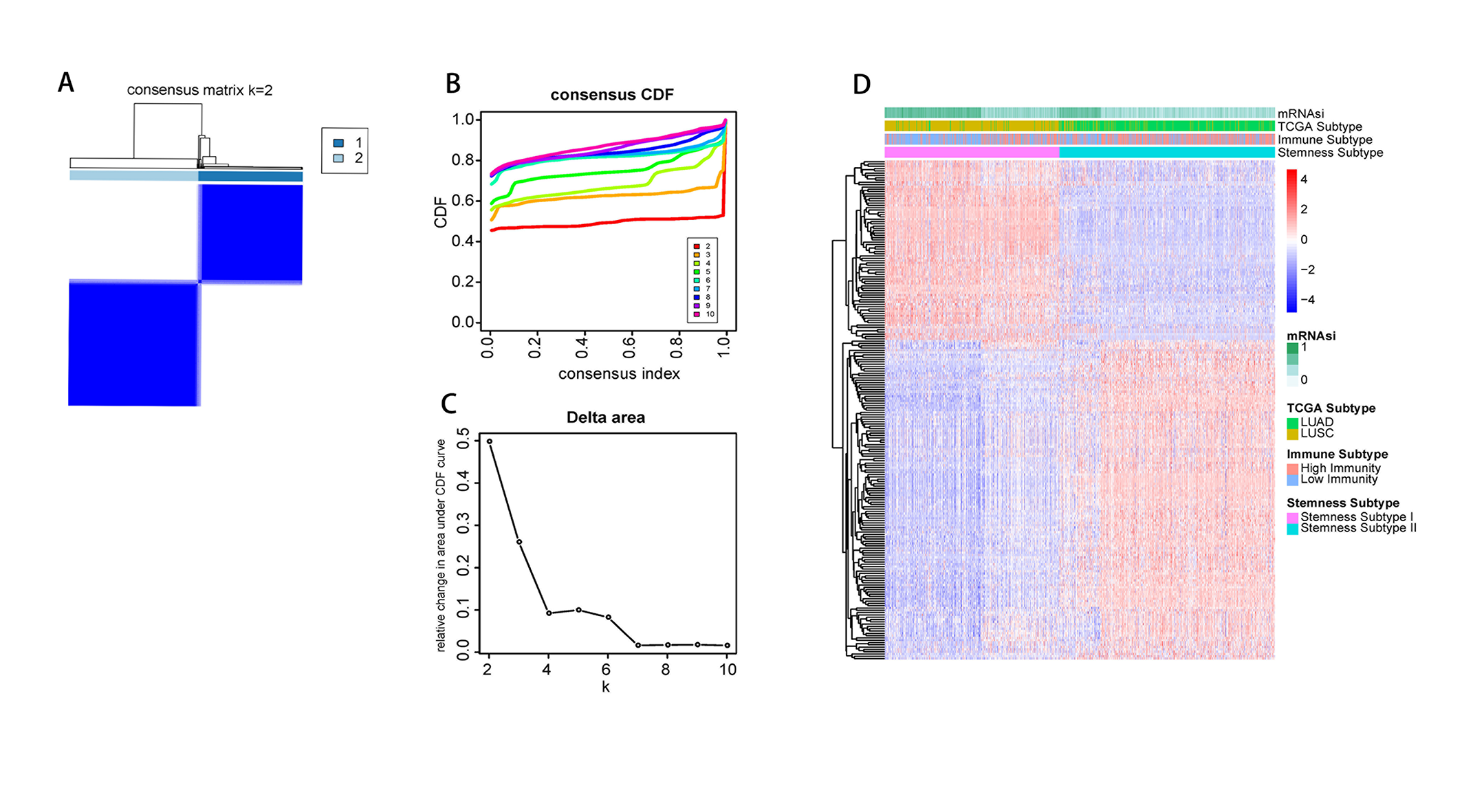

Supplement: Supplementary file 4 — Additional file 4. Figure S3: The process of stemness subtype construction. (A) Consistency matrix plot showing clustering at K=2, which is the optimal number of clusters. (B) CDF plot showing the consensus distributions for each K, K=2-9. (C) Delta area showing the relative change in stability, K=2-9. (D) Heat map showing the expression of 217 DEGs, red indicates high expression and green indicates low expression. The top of the heat map shows the mRNAsi, TCGA subtype and immune subtype for each patient. [file 13287_2023_3406_MOESM4_ESM.tif]

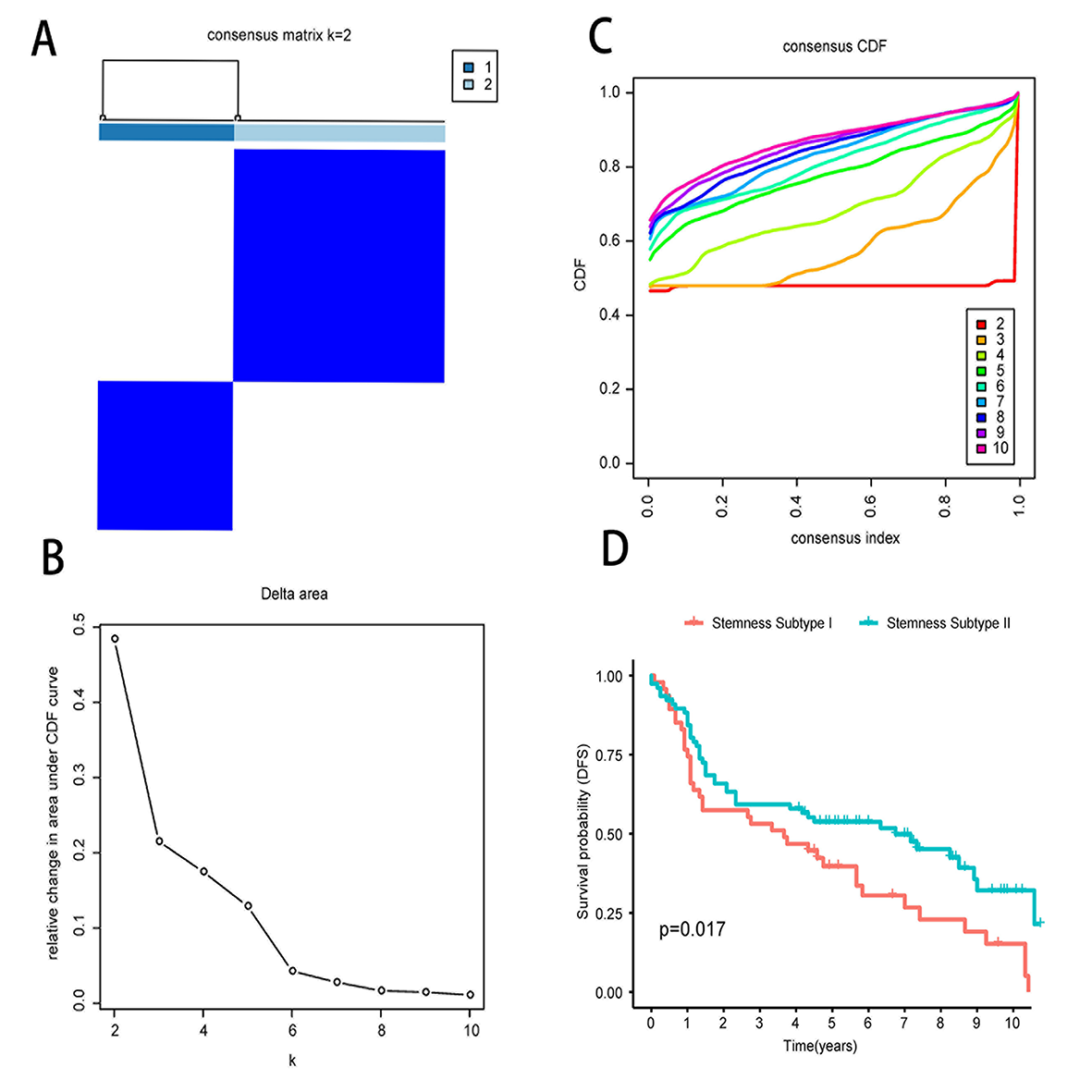

Supplement: Supplementary file 5 — Additional file 5: Figure S4: Identification of a validation set of stemness subtypes based on DEGs. (A) Consistency matrix plot showing the clustering at k=2, which is the optimal number of clusters. (B) CDF plot showing the consensus distribution for each K, k=2-9. (C) Delta area showing the relative change in stability, k=2-9. (D) Survival analysis shows differences in DFS between Stemness Subtype I and Stemness Subtype II. [file 13287_2023_3406_MOESM5_ESM.tif]

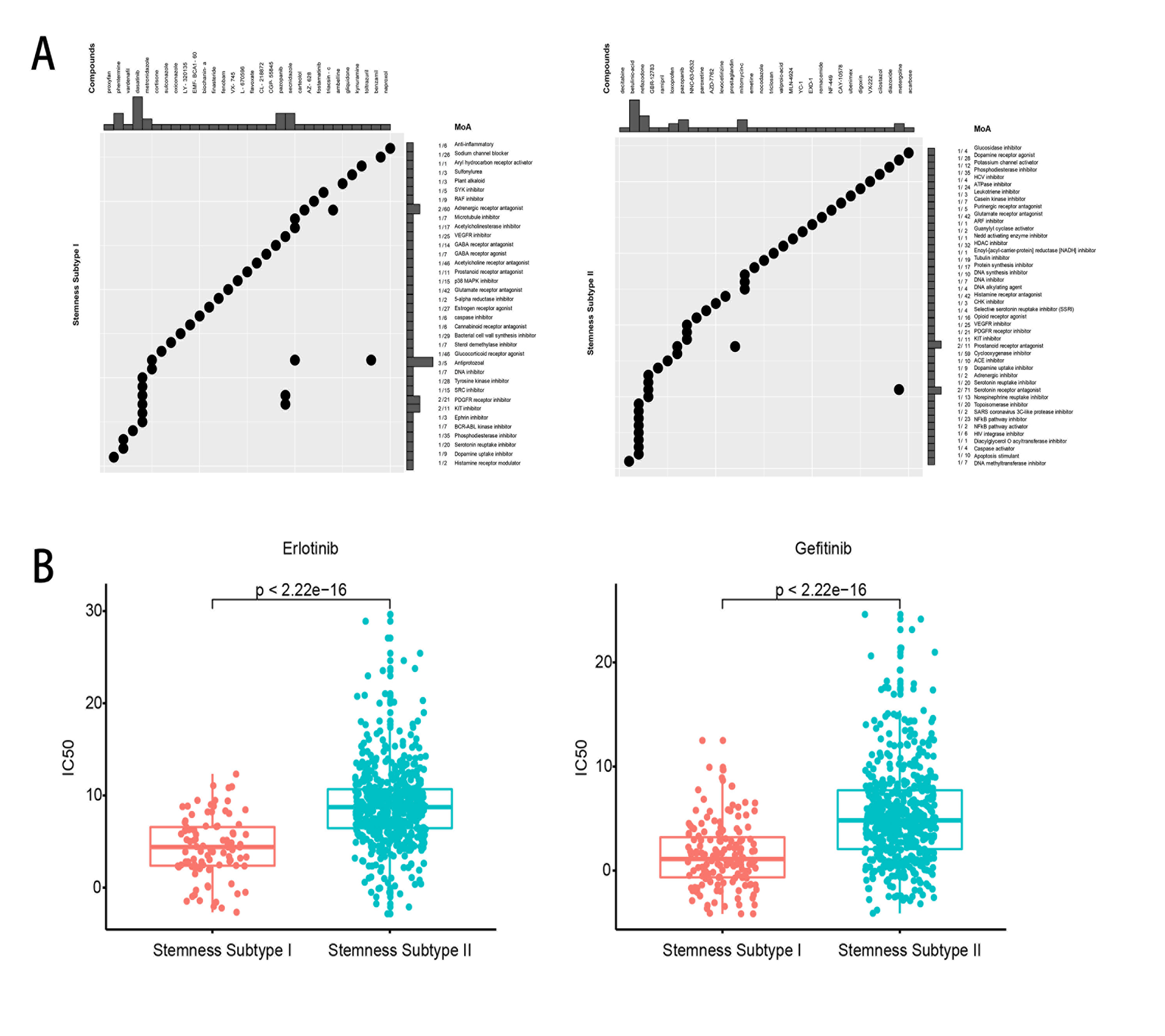

Supplement: Supplementary file 6 — Additional file 6. Figure S5. Stemness subtype chemotherapy sensitivity analysis and potential compound identification. (A) Box plot showing the sensitivity of chemotherapeutic agents between stemness subtypes, red indicates Stemness Subtype I, green indicates Stemness Subtype II. (B) Scatter plot showing the relationship between compounds and MoA in Stemness Subtype I and Stemness Subtype II, rows indicate MoA, columns indicate compounds. [file 13287_2023_3406_MOESM6_ESM.tif]
